# Characterising the bacterial microbiota across the gastrointestinal tracts of dairy cattle: membership and potential function

**DOI:** 10.1038/srep16116

**Published:** 2015-11-03

**Authors:** Shengyong Mao, Mengling Zhang, Junhua Liu, Weiyun Zhu

**Affiliations:** 1 College of Animal Science and Technology, Nanjing Agricultural University, Nanjing 210095, China

## Abstract

The bacterial community composition and function in the gastrointestinal tracts (GITs) of dairy cattle is very important, since it can influence milk production and host health. However, our understanding of bacterial communities in the GITs of dairy cattle is still very limited. This study analysed bacterial communities in ten distinct GIT sites (the digesta and mucosa of the rumen, reticulum, omasum, abomasum, duodenum, jejunum, ileum, cecum, colon and rectum) in six dairy cattle. The study observed 542 genera belonging to 23 phyla distributed throughout the cattle GITs, with the Firmicutes, Bacteroidetes and Proteobacteria predominating. In addition, data revealed significant spatial heterogeneity in composition, diversity and species abundance distributions of GIT microbiota. Furthermore, the study inferred significant differences in the predicted metagenomic profiles among GIT regions. In particular, the relative abundances of the genes involved in carbohydrate metabolism were overrepresented in the digesta samples of forestomaches, and the genes related to amino acid metabolism were mainly enriched in the mucosal samples. In general, this study provides the first deep insights into the composition of GIT microbiota in dairy cattle, and it may serve as a foundation for future studies in this area.

The relationships between gastrointestinal bacterial communities and their mammalian hosts have been shown to provide important benefits to the hosts^1^,^2^,^3^. A classic example of these relationships is found in the rumens of ruminants, where plant digestion enables the conversion of plant fibres into chemical compounds, which subsequently are absorbed and digested by the animal^4^. This process is extremely important to humans, as it enables use of the solar energy stored in plant fibres via their conversion into food products, such as milk and meat. Thus, the microbiota in bovine rumens have been explored extensively. However, the role of microorganisms in other segments of the gastrointestinal tract (GIT), such as the small and large intestine, have received little attention. Consequently, our understanding of the characteristic microbiota in various sections along the GIT is still very limited. Recently, two studies explored the complexity of digesta microbial communities in Brazilian Nelore steers and the composition of digesta- and mucosa-associated microbiota in the GITs of pre-weaned calves^5^,^6^. However, there is little information on the bacterial communities in the GITs of dairy cattle, especially Holstein cattle, which are known today as the world’s highest-production dairy animals.

Although the previously mentioned studies detected the gastrointestinal taxonomic groups and species compositions in steers and pre-weaned calves^5^,^6^, they did not report on the metabolic activities and functions of the microbial communities. The function of the microbiota in communities can be assessed using shotgun metagenomics^7^, but this approach is expensive and challenging to analyse. PICRUSt is an approach for inferring the metagenomes of the closest available whole genome sequences using 16S rRNA gene sequence profiles^8^,^9^; hence, PICRUSt provides a way to predict the changes to microbial function that are likely to be associated with changes in operational taxonomic unit (OTU) abundance detected via 16S sequencing. Therefore, this study first characterised the compositions and phylogenetic distributions of the digesta- and mucosa-associated microbiota within the various compartments of the GITs of Holstein dairy cattle. Then, it applied inferred metagenomics using PICRUSt to investigate functional differences in the gastrointestinal microbial ecosystem.

## Results

### Concentration of volatile fatty acid (VFA) and pH value in the digesta

**Figure 1:**
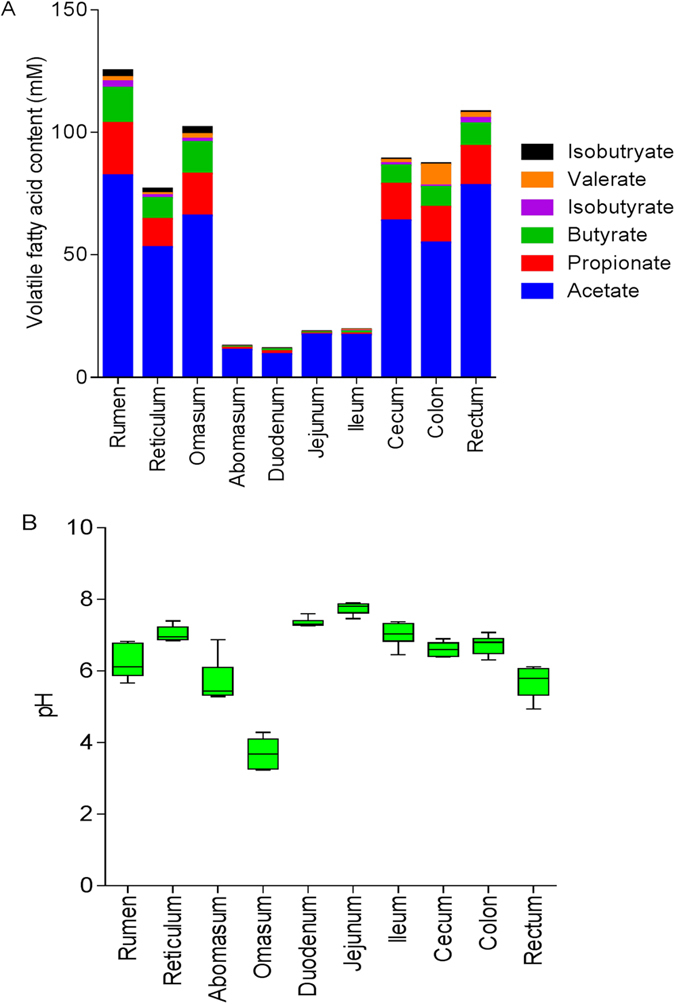
Changes in the pH value and concentrations of volatile fatty acids (VFAs) in the digesta across the gastrointestinal tracts (GITs) of dairy cattle. (**A**) Concentrations of VFAs in the digesta; (**B**) pH value in the digesta across GITs.

In total, concentrations of acetate, propionate, butyrate, isobutryate, valerate and isovalerate in the forestomach (rumen, reticulum and omasum) and the large intestine (cecum, colon and rectum) were significantly higher than in the small intestine (duodenum, jejunum ileum) (Fig. 1A). The pH value in the omasum was the lowest (*p* < 0.05) across the GIT (Fig. 1B).

### Data acquisition and analysis

In this study, a 16S rRNA gene sequence analysis of cattle GIT samples generated 7,560,618 high quality sequences, with an average of 49,832 ± 10,550 sequences per sample. The overall number of OTUs detected by analysis was 6,860, based on a 97% nucleotide sequence identity between reads. To assess whether sampling provided sufficient OTU coverage to describe the bacterial composition of each region accurately, individually based rarefaction curves were generated for each region (Fig. S1). Results showed that the Good’s coverage was greater than 0.97, implying that sampling was sufficient for the samples from all animals (Table S1).

### Diversity, richness and composition of the digesta-associated bacterial communities across cattle GITs

**Figure 2:**
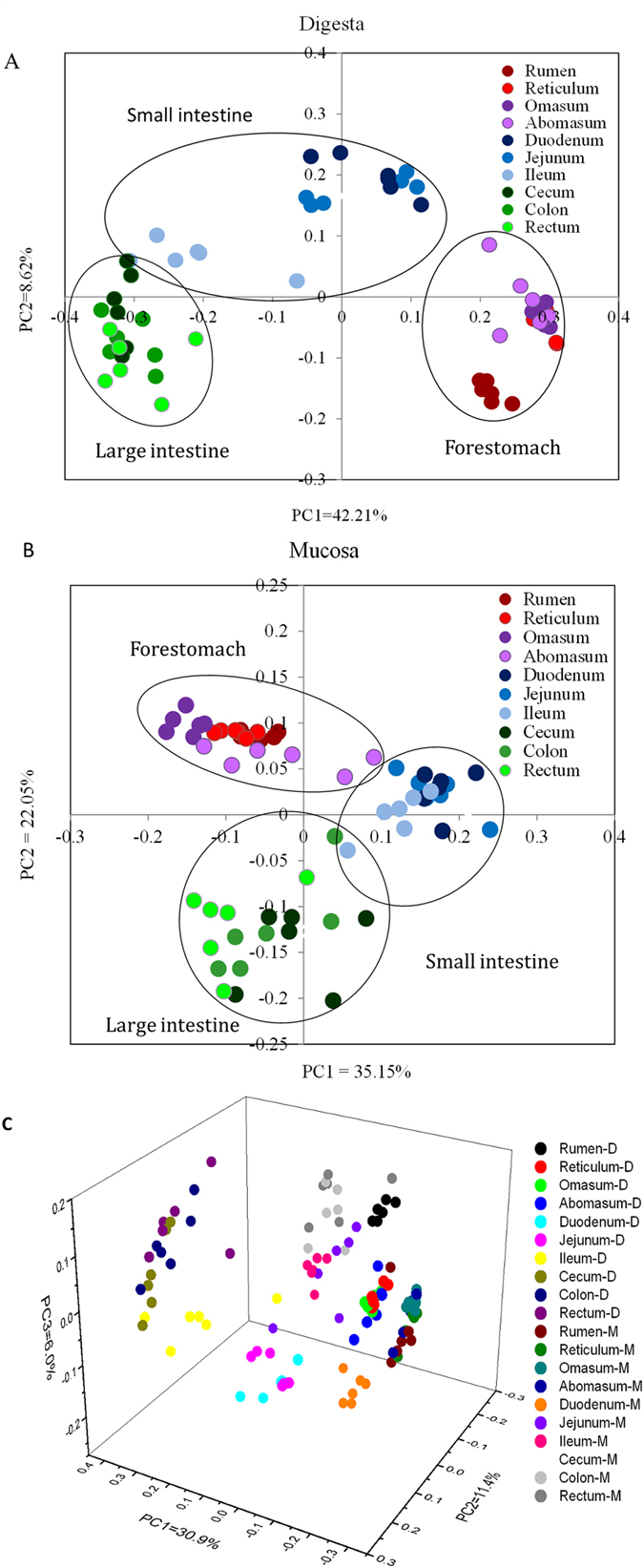
Principal coordinate analysis (PCoA) profile of microbial diversity across all samples using an unweighted UniFrac metric. The percentage of variation explained by PC1 and PC2 are indicated in the axis. (**A**) Unweighted PCoA by digesta bacterial microbiota. (**B**) Unweighted PCoA of mucosal bacterial microbiota. (**C**) Three-dimensional unweighted PCoA of bacterial microbiota across gastrointestinal tracts of dairy cattle. D: digesta samples; M: mucosal samples.

Number of OTUs, Shannon diversity index and richness differed significantly among regions in the digesta samples (Table 1), and the Chao 1 value, number of OTUs and Shannon index in the forestomach were significantly higher than in the small and large intestines (Table 1). When bacterial composition of digesta microbiota among GIT regions was compared using the unweighted UniFrac distance, digesta-associated bacterial communities of the forestomach, small intestine and large intestine were separated spatially from each other (Fig. 2A). An unweighted distance-based analysis of molecular variance (AMOVA) was used to assess the statistical significance of the spatial separation observed among various regions of the principal coordinate analysis (PCoA) plots. Statistically significant dissimilarities were observed across most regions with respect to bacterial diversity, with the exception of similarities between the cecum and the rectum (*p* = 0.052), the colon and the cecum (*p* = 0.179), the rectum and the colon (*p* = 0.485) and the jejunum and the duodenum (*p* = 0.123) (Table S2). In addition, digesta-associated bacterial communities in the forestomach and large intestine samples clustered close to one another, while those from the small intestine samples did not (Fig. 2A).

**Figure 3:**
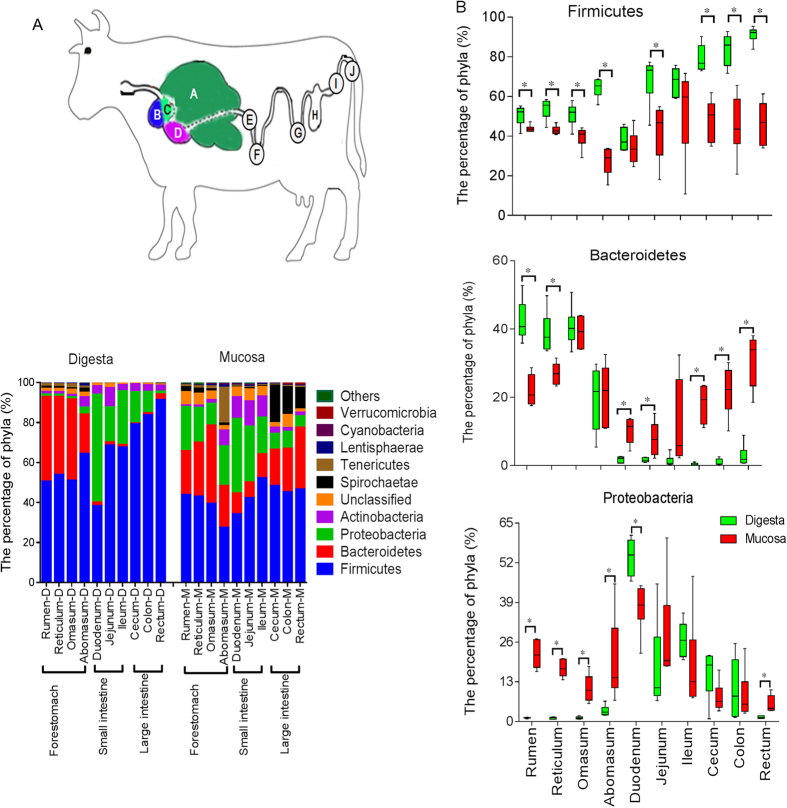
Phylum-level composition. (**A**) Depicted are sampling locations and average relative abundances of phyla across the gastrointestinal tracts (GITs) of dairy cattle. (**B**) Comparison of relative abundances of the three main bacterial phyla found in every sampling site: Proteobacteria, Bacteroidetes and Firmicutes, represented as relative abundances on the Y-axis. Boxes with a star symbol above their whiskers are significantly different between the digesta and its corresponding mucosa in each GIT site at *p* < 0.05 using a t-test analysis. D: digesta samples; M: mucosal samples.

At the phylum level, 21 bacterial phyla were identified in the digesta samples (Table S3). The majority of the sequences obtained belonged to Firmicutes (64.81%), Bacteroidetes (15.06%) and Proteobacteria (13.29%) (Fig. 3A). In addition, only Firmicutes, Bacteroidetes, Proteobacteria, Spirochaetae, Cyanobacteria and Tenericutes were found in all samples. Among the 10 GIT sites, the rumen and abomasum harboured most of the phyla and groups (19 phyla), while the lowest number of phyla was observed in the cecum (12 phyla). When bacterial composition was compared regionally, the phylum Firmicutes dominated all bacterial communities along the GIT except for in the duodenum, where Proteobacteria (45.6%) was predominant. Bacteroidetes was the second most prevalent in the forestomach, while Proteobacteria was the second most prevalent in digesta samples of the jejunum, ileum, cecum and colon (Fig. S2).

**Figure 4:**
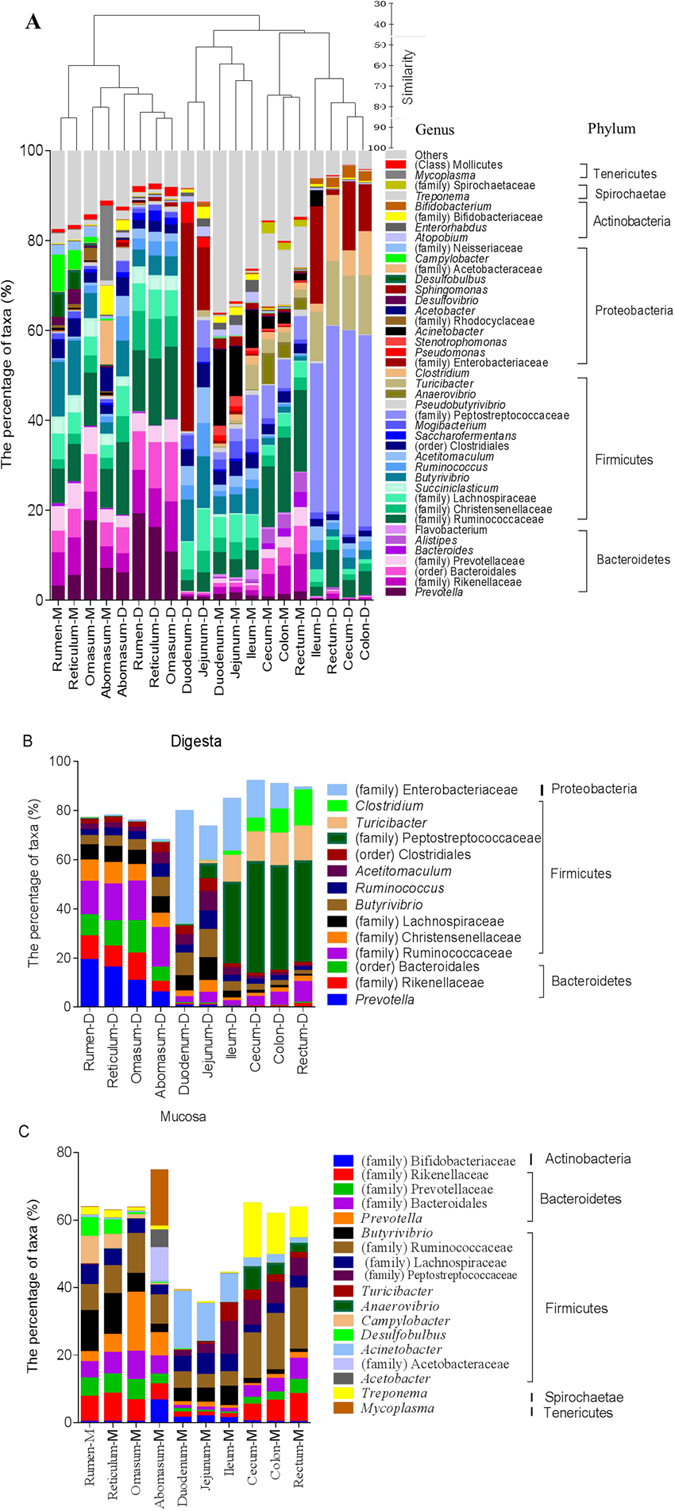
Distribution of genera across the gastrointestinal tracts (GITs) of dairy cattle. (**A**) Spatial distribution of the most abundant taxa (only taxa with relative abundance of ≥2% in at least one GIT region were presented) in the GITs of dairy cattle. The dendrogram shows clustering of the OTU data (Bray-Curtis similarity measure), averaged by sampling sites. (**B**) Comparison of dominant genera (relative abundance of ≥5% in at least one GIT region) in digesta samples across GITs. (**C**) Comparison of predominant genera (relative abundance of ≥5% in at least one GIT region) in mucosal samples across GITs. Taxa that could not be assigned a genus but were present in all samples were displayed using the highest taxonomic level that could be assigned to them. D: digesta samples; M: mucosal samples.

At the genus level, 542 taxa were observed throughout cattle GITs; however, 44.6% of all sequences were not identified at the genus level. For clarity and visualisation purposes, Fig. 4A presents the most abundant taxa (those with a relative abundance of ≥2% in at least one GIT region). Results showed that predominant genera in cattle GITs included *Prevotella*, *Treponema*, *Succiniclasticum, Ruminococcus*, *Acetitomaculum*, *Mogibacterium*, *Butyrivibrio* and *Acinetobacter*, as well as those unclassified derived from Peptostreptococcaceae (family), Ruminococcaceae (family), Enterobacteriaceae (family), Prevotellaceae (family), Clostridiales (order), Rikenellaceae (family) and Bacteroidales (order). In the digesta samples, the dominant taxa, *Prevotella*, unclassified Ruminococcaceae, unclassified Bacteroidales and unclassified Rikenellaceae were enriched significantly in the digesta of forestomach samples (Fig. 4B and Table S4). The unclassified Enterobacteriaceae were enriched [false discovery rate (FDR) <0.001] in the small intestine, cecum and colon, and a large proportion of *Acetitomaculum*, *Ruminococcus* and unclassified Lachnospiraceae were enriched (FDF <0.001) in the jejunum. Proportions of *Butyrivibrio* were significantly higher in the abomasum, duodenum and jejunum than in other GIT regions. The unclassified Peptostreptococcaceae and *Turicibacter* were enriched (FDR <0.001) in the ileum and large intestine, while the *Clostridium* were more enriched in the large intestine (FDR <0.001) than in the forestomach and small intestine.

At the OTU level, the study identified 5,573 OTUs in the digesta samples (Fig. S3). Of them, one OTU, OTU-3825, classified in the family Enterobacteriaceae was the most dominant among the dominant OTUs (representing ≥2% of all sequences in at least one region in all samples), and its abundance was the greatest (FDR <0.001) in the duodenum digesta samples (Fig. S4 and Table S5).

### Diversity, richness and composition of mucosa-associated bacterial communities across cattle GITs

Number of OTUs, Shannon diversity index and richness differed significantly among regions in mucosa samples (Table 1). In the mucosal tissue, the Chao 1 value and the number of OTUs in the forestomach were significantly higher than in the small intestine. No significant differences were observed in the Shannon index among regions, with the exception of the omasum and the cecum (Table 1). When bacterial composition of mucosal microbiota among various GIT regions were compared using unweighted UniFrac distance, the mucosa-associated communities in the forestomach clustered more closely to each other than did other members of the combined large intestinal communities (Fig. 2B). AMOVA testing of mucosal samples revealed that compositions of bacterial communities differed significantly across GIT regions (except for abomasum vs omasum, *p* = 0.082; ileum vs jejunum, *p* = 0.328; cecum vs colon, *p* = 0.949; cecum vs rectum, *p *= 0.224; and colon vs rectum, *p *= 0.585) (Table S2).

At the phylum level, 22 bacterial phyla or groups were identified in mucosal samples (Table S6). The majority of the sequences obtained belonged to Firmicutes (42.22%), Bacteroidetes (21%) and Proteobacteria (17.56%), and Firmicutes, Bacteroidetes, Proteobacteria, Actinobacteria, Tenericutes, Spirochaetae, Cyanobacteria and Lentisphaerae were found in all samples (Fig. 3A). Most of the phyla (18–20/22 phyla) were found among the 10 GITs sites. Firmicutes dominated all mucosa-associated bacterial communities along the GITs except for in the duodenum, where Proteobacteria (37.26%) was predominant. However, relative abundance of the second most predominant phyla varied markedly among mucosal tissue by GIT region (Fig. S2). Bacteroidetes was the second most prevalent phyla in mucosal samples of the reticulum, omasum, abomasum, colon and rectum, whereas Proteobacteria was the second most prevalent in mucosal tissues of the rumen, jejunum and ileum. Firmicutes and Spirochaetae were the second most dominant phyla in mucosal samples of the duodenum and cecum, respectively.

At the genus level, an analysis of the most-abundant taxa by sample revealed that abundances of *Butyrivibrio*, *Campylobacter* and *Desulfobulbus* were enriched significantly in mucosal samples of rumen and reticulum (Fig. 4C and Table S7). In the abomasum, a large proportion of *Mycoplasma*, *Acetobacter*, unclassified Acetobacteraceae and unclassified Bifidobacteriaceae were enriched (FDR < 0.05). In the ileum, *Turicibacter* dominated (FDR < 0.001) compared with the forestomach, duodenum and jejunum samples, and in the cecum, *Anaerovibrio* were enriched significantly compared with the forestomach and small intestine samples. Relative abundance of unclassified Peptostreptococcaceae was higher in the ileum and large intestine (FDR < 0.001) than in the forestomach samples, while *Acinetobacter* was more enriched (FDR < 0.001) in the small intestine than in the forestomach samples. *Treponema* dominated (FDR < 0.001) in the mucosa of the cecum and colon, and *Prevotella* was enriched significantly in the omasum and abomasum. Unclassified bacteria derived from the families Ruminococcaceae and Rikenellaceae were more enriched in the forestomach and large intestine than in the small intestine. In addition, unclassified Bacteroidales were more enriched in the forestomach and large intestine than in the small intestine samples, and unclassified Prevotellaceae dominated (FDR < 0.001) in the forestomach compared with the large and small intestine samples, except for those from the rectum.

At the OTU level, 6,327 OTUs were identified in mucosal samples (Fig. S3). Of them, OTU-332 classified to *Acinetobacter* was the most dominant among the dominant OTUs in the samples, and its abundance was the greatest (FDR < 0.001) in mucosal tissues of the duodenum and jejunum (Table S8 and Fig. S4).

### Comparison of diversity, richness and composition of bacterial microbiota between corresponding digesta and mucosa samples

When alpha diversity indices were compared between mucosal tissue and digesta of each GIT region, OTU values in the ruminal digesta were significantly higher than in the rumen epithelium (Fig. S5A). In contrast, the numbers of OTUs in mucosal tissues of the omasum, cecum, colon and rectum were higher (*p* < 0.05) than in their respective digesta. Mucosal tissues of the reticulum, omasum and large intestine exhibited higher (*p *< 0.05) Chao1 index values than in their respective digesta (Fig. S5B). The Shannon index was significantly higher for digesta of the rumen and reticulum than for their corresponding mucosal tissues, while mucosal tissues of the duodenum, ileum and large intestine had a higher Shannon index than their corresponding digesta (*p*v <v0.05) (Fig. S5C). In addition, a PCoA profile (Fig. 2C) and AMOVA analysis (Table S9) revealed significant differences in composition and structures of bacterial communities between the mucosal tissue and digesta of each GIT region. At the OTU level, the study found 5,040 OTUs across all samples, 533 exclusively in the digesta and 1,287 in the mucosa (Fig. S3).

At the phylum level, the proportion of Firmicutes in the digesta of the forestomach, jejunum and large intestine was significantly higher than in their corresponding mucosal tissues (Fig. 3B). The abundance of Bacteroidetes was higher (FDR < 0.001) in the digesta of the rumen and reticulum, while it was lower (FDR < 0.001) in the digesta of the duodenum, jejunum and large intestine when compared with their corresponding mucosal tissues. Mucosal tissues of the forestomach and rectum presented higher (FDR < 0.001) proportions of Proteobacteria than their corresponding digesta samples, while digesta of the duodenum showed a comparatively higher proportion of Proteobacteria.

At the genus level, in the rumen, the proportions of *Prevotella*, unclassified Ruminococcaceae, unclassified Rikenellaceae, unclassified Christensenellaceae and unclassified Bacteroidales were significantly higher in digesta samples than in mucosal samples, while rumen mucosa presented higher (FDR < 0.001) percentages of *Butyrivibrio*, unclassified bacteria, *Desulfobulbus* and *Campylobacter* (Fig. S6A). In the small intestine, luminal samples presented a significantly higher abundance of unclassified Enterobacteriaceae and a lower abundance of *Acinebacte*r compared with corresponding mucosal samples (Figs S6B, S6C and S6D). In the large intestine, unclassified Peptostreptococcaceae, *Turicibacter* and *Clostridium* were dominant in the lumen (Figs S6E, S6F and S6G), while *Treponema* and unclassified Ruminococcaceae were more abundant in the mucosa.

### Total bacterial populations in the GIT of dairy cattle

Total bacterial populations in various GIT regions were estimated with a real-time PCR analysis by measuring the total copy number of bacterial 16S rRNA genes. Regional sites affected bacterial density of dairy cattle significantly (Table 2). Higher digesta-associated bacterial numbers (*p* < 0.05) were observed in the colon and omasum, and higher mucosa-associated bacterial numbers were present in the rumen and omasum (Table 2). In addition, mucosa-associated bacterial densities in the jejunum, ileum, cecum, colon and rectum were significantly lower than in their corresponding digesta (Table 2). However, digesta-associated bacterial numbers in the duodenum were lower than mucosa-associated bacterial densities (*p* = 0.002).

### Predicted molecular functions of bacterial microbiota

**Figure 5:**
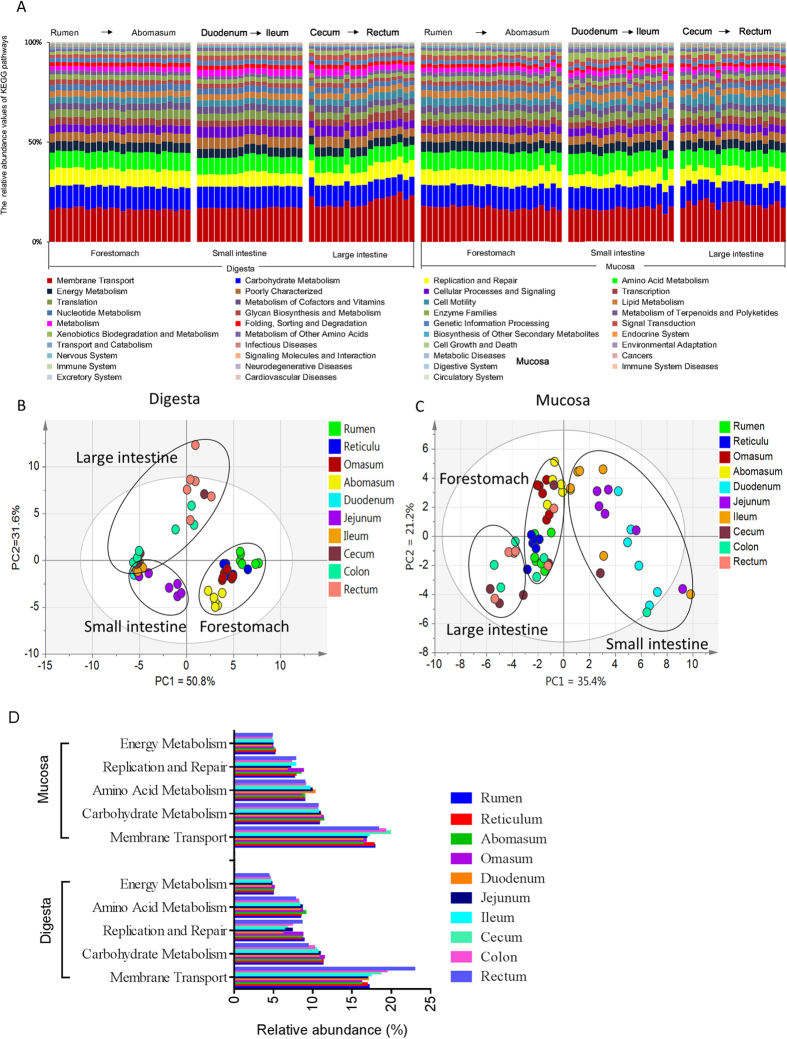
Metagenomic functional predictions for samples. (**A**) Variations in KEGG metabolic pathways in functional bacterial communities throughout the gastrointestinal tracts (GITs) of dairy cattle. (**B**) Principal coordinate analysis (PCA) of microbial functional diversity across all digesta samples using the relative abundances of functional pathways. (**C**) PCA of microbial functional diversity across all mucosal samples. (**D**) Comparisons of the five predominant gene pathways of the bacterial microbiota throughout the GITs of dairy cattle.

To gain insight into the molecular functions of bacterial microbiota across cattle GITs, we used PICRUSt to predict the metagenomic contribution of the communities observed. PICRUSt predicts metagenomic potential by imputing the available annotated genes within a known sequenced database, such as the Kyoto Encyclopaedia of Genes and Genomes (KEGG) and the Clusters of Orthologs Groups (COGs) catalogue, based on the presence/absence of OTUs in a 16S rRNA survey. With PICRUSt, one can calculate nearest sequenced taxon index (NSTI), which measures how closely related the average 16S rRNA sequence in an environmental sample is to an available sequenced genome. When this number is low, PICRUSt is likely to perform well in predicting the genomes of the organisms in an environmental sample. This study used the KEGG database to match the chosen reference OTUs, and the average NSTI for the 120 samples was 0.075. This low NSTI metric suggests that PICRUSt may perform well when predicting the molecular function of microbial communities in the GITs of dairy cattle. Using PICRUSt as a predictive exploratory tool, the present study inferred that 39 gene families were identified in the digesta and mucosa samples (Fig. 5A). A principal component analysis (PCA) on the relative abundance values of the KEGG pathways represented from the digesta and mucosal microbiota showed a clear distinction between the clustering of the intestine digesta and that of the forestomach samples (Fig. 5B). In the mucosal tissues, PCA observed a separate clustering of the forestomach and intestine samples, except for those of the rectum (Fig. 5C).

Of the 39 gene families, the majority of the genes belonged to membrane transport (17.82% in digesta-associated microbiota and 17.61% in mucosa-associated microbiota, respectively), carbohydrate metabolism (10.68% in digesta, 10.79% in mucosa), amino acid metabolism (8.36% in digesta, 9.13% in mucosa), replication and repair (7.70% in digesta, 7.62% in mucosa) and energy metabolism (4.69% in digesta, 4.84% in mucosa). Of the 39 gene families, 33 gene families in the mucosa-associated microbiota had significantly different abundances among GIT regions (Table S10), and the prevalence of 37 gene families in the digesta-associated microbiota was significantly different among GIT regions (Table S11). Of the five predominant gene families mentioned earlier, in the digesta samples, the relative abundances of the genes involved in carbohydrate metabolism and replication and repair in the microbiota of forestomach samples were significantly higher than in the microbiota of the cecum and colon (Fig. 5D and Table S12). The abomasum had the highest (FDR < 0.001) abundance of genes involved in amino acid metabolism, while the proportion of gene families involved in amino acid metabolism in the rectum was the lowest (FDR < 0.001) throughout the GIT (Table S12). In mucosal samples, there was a notable enrichment (FDR < 0.001) of genes related to amino acid metabolism in the duodenum when compared with those in the forestomach and large intestine. The microbiomes in the abomasum and the omasum were significantly enriched in categories associated with carbohydrate metabolism (Table S12).

Of the five predominant gene families, when compared with corresponding digesta samples, the abundance of genes related to membrane transport was significantly higher in the mucosa of the reticulum, while it was lower (FDR < 0.05) in the mucosa of the duodenum and rectum (Fig. S7A). The proportion of genes involved in carbohydrate metabolism was higher (FDR < 0.001) in the digesta of rumen and reticulum compared with their corresponding mucosa, while it was significantly lower in the digesta of duodenum and rectum compared with their corresponding mucosal samples (Fig. S7B). Genes related to replication and repair were significantly enriched in abundance in the ruminal and reticulum digesta-associated microbiota compared with the mucosa samples (Fig. S7C). Genes involved in amino acid metabolism were higher in percentage in mucosa-associated microbiota (except for in the abomasum, cecum and colon) (FDR < 0.001) than they were in their corresponding digesta samples (Fig. S7D). In addition, genes related to energy metabolism were significantly higher in mucosa of the rumen, reticulum, cecum, colon and rectum compared with their corresponding digesta samples (Fig. S7E).

## Discussion

This study characterised the compositions and phylogenetic distributions of digesta- and mucosa-associated microbiota in the GITs of Holstein dairy cattle. Results showed significant differences in bacterial richness and diversity, as indicated by the Chao 1 values and Shannon index, among GIT regions (Table 1), indicating that GIT region exhibit strong determinants of the microbial community composition in dairy cattle. These results may be explained by the difference in the luminal pH values of different regions, as well as by the gut motility, redox potential, nutrient supplies and host secretions^10^. In addition, results from PCoA profiling and AMOVA analysis revealed significant differences among sections of the GIT, implying that GIT region is strongly determinant of microbial community structure.

Overall, findings of the present study revealed that the taxonomic groups represented within cattle GIT were Firmicutes, Bacteroidetes and Proteobacteria: These varied considerably among regions in abundance and in the number of genera composing them (Fig. 4A). In general, the digesta-associated microbiota of the forestomach exhibit greater relative abundances of Firmicutes and Bacteroidetes, whereas the small intestine and large intestine, except for the rectum, show higher relative abundances of Firmicutes and Proteobacteria (Fig. S2). Interestingly, the phylum Firmicutes, which was prominent in digesta-associated microbiota of the large intestine, was composed mainly of the genera *Clostridium*, *Turicibacter* and unclassified Peptostreptococcaceae (Table S4), which together reached up to 70% of the total reads in some samples. However, the dominant taxa belonging to Firmicutes in the digesta-associated forestomach microbiota were unclassified Ruminococcaceae, unclassified Rikenellaceae, unclassified Christensenellaceae and unclassified Lachnospiraceae (Table S4), which have been detected widely in the rumen and implicated as playing an important role in degradation of starch and fibre^11^. The fact that members of Ruminococcaceae, Christensenellaceae and Lachnospiraceae dominated in the forestomach while *Turicibacte*r, *Clostridium* and members of Peptostreptococcaceae were enriched in the large intestine revealed significant differences in composition of the predominant microbial communities between the forestomach and large intestine.

The phylum Bacteroidetes was significantly less abundant in digesta-associated microbiota of the small and large intestine, whereas in the rumen, it was prominent and mainly composed of the genus *Prevotella* (Table S4), which reached up to 19.08% of the total reads in some digesta samples. This finding was consistent with a previous study by Stevenson and Weimer^12^, who reported that *Prevotella* were the most abundant (making up 17–50% of total bacterial reads) of all genera identified in rumen samples from lactating cows. High abundance of this genus in the rumen is thought to relate to the high genetic variability of this genus, which enables its members to occupy various ecological niches within the rumen^13^. However, the exact mechanism explaining the result that the genus *Prevotella* was less abundant in digesta-associated microbiota of small and large intestines is not clear yet, and further studies are required.

The phylum Proteobacteria was significantly less abundant in digesta-associated microbiota of the forestomach, whereas its abundance was significantly increased in digesta samples of the small and large intestines, except for the rectum (Fig. 3A). The high abundance of Proteobacteria in digesta of the small intestine was attributed mainly to OTUs representing unclassified Enterobacteriaceae (Table S5). Unclassified Enterobacteriaceae refers to bacterial genera encompassed by the Enterobacteriaceae family, about the function of which little is known yet^14^. As these unclassified gut bacteria have not yet been evaluated functionally, the cause of the increase in abundance of unclassified Enterobacteriaceae in the digesta of the small intestines is not entirely clear, and future studies are needed to clarify these issues.

Gastrointestinal mucosa-associated microbiota could play important biological roles due to their close proximity to the animal host, but knowledge of their composition in cattle still is limited to calves^6^,^15^. The mucosal microbiota of calves has previously been investigated using culture-based methods and culture-independent DNA techniques^6^,^16^, and these studies revealed the presence of mucosa-associated bacterial communities with bacterial species that differed from those associated with the digesta of calves. In the present study, our data revealed significant differences in the diversity index of mucosa-associated bacteria communities among most GIT regions (Table 1). In addition, PCoA profiling and AMOVA analysis revealed significant differences in composition and structure of mucosa-associated bacteria communities among GIT sections. Thus, the present study has provided further evidence of mucosa-associated bacterial communities along the GITs of dairy cattle, which appears to support previously reported trends regarding gastrointestinal region-specific microbiota.

Among the regions of the GIT, in contrast to the rumen epithelium, the mucosa-associated bacterial microbiota of the small and large intestines has been studied infrequently. The present study highlighted the dominant presence of the aerobic bacterial genus *Acinetobacter* in small intestine mucosa. This finding is in accordance with previous works showing the presence of oxygen at the apical surface of the intestinal epithelial cells^17^, representing a possible mechanism of exclusion of strictly anaerobic, extremely oxygen-sensitive microorganisms^18^. In addition, the present study found a higher abundance of *Butyrivibrio* (belonging to Firmicutes) in the rumen and reticulum mucosal samples across the GITs of dairy cattle. As the mucosal butyrate producers release butyrate close to the epithelium, species of *Butyrivibrio* may enhance butyrate bioavailability for the host, which may be particularly useful in proliferating rumen and reticulum epithelium^19^,^20^. In addition, the present study’s finding of the enrichment of the genus *Treponema* (belonging to Spirochaetae) in large intestine mucosa may have health implications. Previous studies revealed that *Treponema* spp., well adapted to oxidative stress^21^, are involved in a number of diseases of the skin or mucus membranes in several mammals^22^. Additionally, *Treponema* spp. are believed to be associated with ulcerative mammary dermatitis and bovine digital dermatitis in cattle and contagious ovine digital dermatitis in sheep^23^,^24^,^25^. Thus, enhanced *Treponema* in large intestine mucosa could have deleterious effects on hindgut health. Altogether, findings of the present study revealed that abundances of dominant genera in mucosa-associated microbiota varied considerably among GIT regions, and uniform distribution of the attaching bacterial compositions along the rumen, small intestine and hindgut is likely due to host-bacterium interactions in the mucosa.

Consistent with the findings in bovine calves using denaturing gradient gel electrophoresis, clone library and pyrosequencing^6^,^15^, the present study revealed a higher proportions of the predominant taxa *Prevotella*, unclassified Ruminococcaceae, unclassified Enterobacteriaceae and unclassified Peptostreptococcaceae in the digesta-associated microbiota (Fig. S6) and a larger percentage of *Butyrivibri*o, *Acinetobacter* and *Treponema* in the mucosa. Previous studies revealed that the genus *Prevotella*, unclassified Ruminococcaceae and unclassified Peptostreptococcaceae might play important roles in feed digestion^26^,^27^ and that members of the genera *Butyrivibrio*, *Acinetobacter* and *Treponema* were more involved in epithelium proliferation and diseases^6^,^28^. These findings imply that sampling sites show strong determinants of microbial community structure and function of bacterial communities among regions.

The cattle gastrointestinal microbiome presents many physiological functions that are lacking in the host, and therefore, they can be considered essential to cattle life. To determine the potential functions of the gastrointestinal microbiota in the samples, the present study used PICRUSt to infer putative metagenomes from 16S rRNA gene profiles^8^. The study inferred that the most abundant functional categories were those corresponding to the functions of carbohydrate metabolism, amino acid metabolism, membrane transport and replication and repair. This is consistent with the general metabolic functions (such as carbohydrate, protein and amino acid metabolism) being essential for microbial survival^29^,^30^, and it is in line with the observations of other metagenomic studies in mice and humans^31^,^32^,^33^. Findings of the present study revealed significant differences in bacterial function among regions across the GIT (Fig. 5B,C). For example, genes relating to carbohydrate metabolism were more abundant in the digesta-associated microbiota of the forestomach than in that of the hindgut. The increased occurrence of these genes mirrors the increase in sequences affiliated with carbohydrate degradation, as discussed above, supporting the importance of these bacteria within the forestomach. In addition to these findings, the present study inferred that genes associated with amino acid metabolism were more enriched in the mucosa-associated microbiota of most GIT regions than in their corresponding digesta (Fig. S7D). One possibility is that the mucosal tissue provides a decreased supply of carbohydrates and that bacteria may derive energy from amino acid fermentation^15^. In addition, these results imply that the mucosal microbiota may be more necessary to amino acid degradation.

In this study, next-generation sequencing on the Miseq platform and bioinformatics analyses were performed to investigate the GIT bacterial microbiota of dairy cattle. Results revealed that microbial communities of dissimilar composition and metabolic function occupied different regions within the GIT ecosystems of dairy cattle, while there were some shared traits across all microbiota. The forestomach, small intestine and large intestine were characterised by a specific microbial community, likely shaped by different physicochemical conditions, such as pH value. Specifically, the taxonomic shift between digesta- and mucosa-associated bacteria and from the rumen to the rectum within cattle GITs supports the assumption that digesta-associated microbiota might play an important role in feed digestion, while the mucosa-adherent microbiome may be involved in epithelium proliferation and diseases, indicating that the structure and function of mucosal and ruminal bacterial communities are distinct. Similarly, the present study inferred that genes related to amino acid metabolism were overrepresented in mucosal samples. These findings indicate that the microbial community associated with mucosa may be more necessary to amino acid degradation. In general, this research has revealed partially the high level of heterogeneity in species composition and functional capacities of microbial assemblages across the bacterial ecology system of the GIT, and these findings can be used to potentially modulate gastrointestinal microbiota and improve health, nutrient use and milk production in dairy cattle.

## Materials and Methods

### Ethical approval

The experimental design and procedures were approved by the Animal Care and Use Committee of Nanjing Agricultural University, in compliance with the Regulations for the Administration of Affairs Concerning Experimental Animals (The State Science and Technology Commission of P. R. China, 1988).

### Animals and sample collection

The study used six Holstein dairy cattle aged five years (body weight: 607 ± 55.6 kg; milk yield = 29.72 ± 4.7 kg/day, 140 to 189 days in lactation). The cows’ diets were formulated to meet or exceed the energy requirements (at 24 kg/day dry matter intake) of Holstein cattle yielding 35 kg of milk/day with 3.50% milk fat and 3.10% true protein (Table S13). Diets were fed ad libitum as a total mixed ration to avoid the selection of dietary components. The cattle were fed at 07:00 and 18:00 h, one-half of the allowed daily ration at each feeding. The experimental period was 84 d; the first 83 d were used for diet adaptation and the last day was used for measurements. Throughout the experimental period, the cattle were housed in tie stalls and fed ad libitum to assure 5% orts, and they were given free access to fresh water during the trial.

On day 84, the cattle were slaughtered, and mucosal tissue and digesta samples were collected from the rumen, reticulum, omasum, abomasum, duodenum, jejunum, ileum, cecum, colon and rectum. Mucosal tissue samples (60 total) were rinsed 3 times with sterile, phosphate-buffered saline (PBS) (pH 7.0) to remove the digesta, cut into 4–5 mm^2^ and immediately frozen in liquid nitrogen. The digesta of the rumen, reticulum, omasum, abomasum, duodenum, jejunum, ileum, cecum, colon and rectum were homogenised, separately. The pH values of the contents of each GIT segment were determined immediately using an Accumet gel-filled polymer-body combination pH electrode (Fisher Scientific; Fairlawn, NJ, USA). Then, the homogenised digesta from each GIT segment were sampled (60 total) and immediately frozen in liquid nitrogen. The remaining samples were centrifuged immediately at 2,000 × g, and the supernatants were stored at −20 °C until they were analysed for VFA. The amount of VFA was measured using capillary column gas chromatography (GC-14B, Shimadzu; capillary column: 30 m × 0.32 mm × 0.25 mm film thickness; column temperature = 110 °C, injector temperature = 180 °C, detector temperature = 180 °C)^27^.

### DNA extraction, PCR amplification, illumina MiSeq sequencing and sequencing data processing

For DNA extraction, three gram (wet weight) of homogenised samples (120 total) of digesta and mucosal tissue from each GIT segment of each cow was used. DNA was extracted by a bead-beating method using a mini-bead beater (Biospec Products; Bartlesville, USA), followed by phenol-chloroform extraction^27^. The solution was precipitated with ethanol, and the pellets were suspended in 50 μL of Tris-EDTA buffer. DNA was quantified using a Nanodrop spectrophotometer (Nyxor Biotech; Paris, France) following staining using a Quant-it Pico Green dsDNA kit (Invitrogen Ltd.; Paisley, UK). DNA samples were stored at −80 °C until further processing.

The V3-V4 regions of the bacteria 16S rRNA gene were amplified by PCR (95 °C for 2 min, followed by 25 cycles at 95 °C for 30 s, 55 °C for 30 s, 72 °C for 30 s and a final extension at 72 °C for 5 min) using primers 338F (5′-barcode- ACTCCTRCGGGAGGCAGCAG)-3′ and 806R (5′-GGACTACCVGGGTATCTAAT-3′), where barcode is an eight-base sequence unique to each sample. PCR reactions were performed in a triplicate 20 μL mixture containing 4 μL of 5 × FastPfu Buffer, 2 μL of 2.5 mM dNTPs, 0.8 μL of each primer (5 μM), 0.4 μL of FastPfu Polymerase and 10 ng of template DNA. Amplicons were extracted from 2% agarose gels and purified using the AxyPrep DNA Gel Extraction Kit (Axygen Biosciences; Union City, CA, USA) according to the manufacturer’s instructions and quantified using QuantiFluor™ -ST (Promega; USA). Purified amplicons were pooled in equimolar and paired-end sequenced (2 × 250) on an Illumina MiSeq platform according to standard protocols^34^.

Raw fastq files were de-multiplexed and quality-filtered using QIIME (version 1.70)^35^, with the following criteria: 1) The 250 bp reads were truncated at any site receiving an average quality score < 20 over a 10 bp sliding window, discarding the truncated reads that were shorter than 50 bp; 2) Exact barcode matching, 2 nucleotide mismatch in primer matching, reads containing ambiguous characters were removed; 3) Only sequences that overlapped longer than 10 bp were assembled according to their overlap sequence. Reads that could not be assembled were discarded. OTUs were clustered with a 97% similarity cut-off using UPARSE (version 7.1 http://drive5.com/uparse/), and chimeric sequences were identified and removed using UCHIME^36^. The most abundant sequences within each OTU were designated as ‘representative sequences’ and aligned against the core set of Greengenes 13.5^37^ using PYNAST^38^ with the default parameters set by QIIME. A PH Lane mask supplied by QIIME was used to remove hypervariable regions from the aligned sequences. FASTTREE^39^ was used to create a phylogenetic tree of the representative sequences. Sequences were classified using the Ribosomal Database Project (RDP) classifier with a standard minimum support threshold of 80%^40^. Sequences identified as chloroplasts or mitochondria were removed from analysis. Community diversity was estimated using the ACE, Chao1 and Shannon indices. The unweighted UniFrac distance method was used to perform a principal coordinate analysis^41^, and an unweighted distance-based analysis of molecular variance (AMOVA) was conducted to assess significant differences among samples using the programme MOTHUR v.1.29.0^42^.

### Predicted molecular functions based on 16S rRNA data using PICRUSt

The present study used PICRUSt^8^ to predict the molecular functions of each sample based on 16S rRNA data. PICRUSt is a bioinformatics tool that uses marker genes, in this case 16S rRNA, to predict the gene functional content of microorganisms. These predictions are pre-calculated for genes in databases including KEGG^43^ and COGs. The present study used the KEGG database and performed closed reference OTU picking using the sampled reads against a Greengenes reference taxonomy (Greengenes 13.5) using the pick_closed_reference_OTU.py script in QIIME^35^. The 16S copy number was normalised using the normalize_by_copy_number.py script, molecular functions were predicted using the predict_metagenomes.py and data were summarised into KEGG pathways using the categorize_by_function.py script, all included in PICRUSt^8^. In addition, PICRUSt calculated the NSTI to quantify dissimilarity between reference genomes and the predicted metagenome presented here. The difference in predicted molecular functions of bacterial communities among GIT regions was determined by a PCA using the SIMCA-P (11.5) software package (Umetrics; Umea, Sweden).

### Real-time PCR analysis of total bacterial populations

Copy numbers of the 16S rRNA genes associated with GIT bacteria were measured using real-time PCR. Primer pairs for the total bacteria were Total bacF (5′-CCATTGTAGCACGTGTGTAGCC-3′) and Total bacR (5′-CGGCAACGAGCGCAACCC-3′), which were reported by Hook *et al.*^44^. PCR reactions were performed in triplicate with SYBR Green PCR Mastermix (Applied Biosystems; Foster City, CA, USA). The total volume of each reaction solution contained 10μl Fast SYBR® Green Master Mix (Applied Biosystems), 0.5 μL of each primer (20 pmol.μL^−1^), 8 μl of nuclease-free water and 1 μL of the template (10 ng.μL^−1^), as described previously^45^. Amplification was carried out using the following programme: 95 °C for 10 min for the initial denaturation and then 40 cycles of 95 °C for 20 s followed by annealing/extension for 1 min at 62 °C. A standard curve was constructed using serial dilutions of plasmid DNA containing the 16S rRNA gene sequence of *Streptococcus bovis*. Real-time PCR efficiency ranged from 90 to 103%, and negative controls without the DNA template were run with every assay to assess overall specificity.

Copy numbers for each standard curve were calculated based on the following equation: (NL × A × 10^−9^)/(660 × n), where NL was the Avogadro constant (6.02 × 10^23^), A was the molecular weight of DNA molecules (ng) and n was the length of amplicon (bp). The copy number of the 16S rRNA genes for total bacteria per gram of sample was calculated using the following equation, as previously reported^15^: (QM × C × DV)/(S × W), where QM was the quantitative mean of the copy number, C was the DNA concentration of each sample (ng.μL^−1^), DV was the dilution volume of extracted DNA (μL), S was the DNA amount subjected to analysis (ng) and W was the sample weight subjected to DNA extraction (g).

### Statistical analysis

GIT region effects on gastrointestinal pH, VFA levels, bacterial prevalence and relative abundance values of the KEGG pathways were analysed using a one-way ANOVA with Dunnett’s post-hoc comparison procedure of SPSS (SPSS v.16, SPSS Inc.; Chicago, IL, USA) according to the following equation: Y_ij_ = μ + G_i_ + e_ij_, where Y_ij_ was the observation (gastrointestinal pH, VFA levels, the relative abundance of a given bacterial phyla, genera, or specie (in %) or the relative abundance values of the KEGG pathways (%)), μ was the overall mean, G_i_ was the gut region effect (I = 10), and e_ij_ was the residual error. An independent t-test was used to test the sample type (mucosal tissue vs. digesta) effects on bacterial prevalence or relative abundance values of the KEGG pathways. All *p*-values from the ANOVA and independent t-test of relative abundance of bacterial taxa and the KEGG pathways were corrected for an FDR of 0.05 using the Benjamini-Hochberg method^46^. FDR-corrected *p*-values below 0.05 (FDR < 0.05) were considered significant.

## Additional Information

**How to cite this article**: Mao, S. *et al.* Characterising the bacterial microbiota across the gastrointestinal tracts of dairy cattle: membership and potential function. *Sci. Rep.* **5**, 16116; doi: 10.1038/srep16116 (2015).

## Supplementary Material

Supplementary Information

## Figures and Tables

**Table 1:** Valid sequences and alpha diversity.

Regions	Digesta				Mucosa			
	Reads	OTUs	Chao value	Shannon index	Reads	OTUs	Chao value	Shannon index
Rumen	47758	2390.2^a^	3050.5^a^	6.26^a^	50462	1976.5^abc^	2628.5^abcd^	5.47^abc^
Reticulum	45589	2109.0^ab^	2537.8^b^	6.19^a^	52697	2199.7^ab^	2779.5^ab^	5.86^ab^
Omasum	44329	2026.0^b^	2475.3^b^	6.08^a^	44820	2388.3^a^	2914.3^a^	6.21^a^
Abomasum	47004	2156.7^ab^	2672.8^ab^	6.02^a^	57897	2314.0^ab^	2731.3^ab^	5.38^bc^
Duodenum	41368	1204.5^cd^	1819.8^cd^	3.49^c^	64588	1293.0^e^	1923.2^e^	5.36^bc^
Jejunum	40972	1365.8^c^	2009.8^c^	4.79^bc^	52848	1388.7^de^	2159.2^de^	5.27^bc^
Ileum	48404	1090.2^cd^	1668.2^cde^	3.50^c^	53495	1295.0^e^	1982.0^e^	5.12^bc^
Cecum	49399	950.3^d^	1378.3^e^	3.13^d^	54466	1536.7^cde^	2173.8^cde^	5.07^c^
Colon	51015	1021.8^d^	1430.7^de^	3.24^c^	55301	1616.3^cde^	2235.3^bcde^	5.40^bc^
Rectum	47772	1086.2^cd^	1526.2^de^	3.49c	56682	1845.5^bcd^	2346.8^abcde^	5.63^abc^
SEM	1006	71.959	77.091	0.175	1513	60.693	56.087	0.063
*P* value	0.386	<0.001	<0.001	<0.001	0.332	<0.001	<0.001	<0.001

Note. Means within the same column with different subscripts are significantly different from one another.

**Table 2:** Total bacterial density (numbers per gram wet weight) throughout the gastrointestinal tracts of dairy cattle*.

Regions	Digesta	Mucosa tissue	*P* value‡
Rumen	(7.79 ± 1.52) × 10^10c^	(5.84 ± 0.64) × 10^10a^	0.575
Reticulum	(7.50 ± 1.1) × 10^10c^	(3.51 ± 0.49) × 10^10ab^	0.136
Omasum	(6.81 ± 0.43) × 10^11ab^	(6.51 ± 0.95) × 10^10a^	<0.001
Abomasum	(3.05 ± 1.32) × 10^10c^	(3.42 ± 0.75) × 10^10b^	0.645
Duodenum	(1.14 ± 0.20) × 10^9d^	(4.93 ± 0.39) × 10^9b^	0.002
Jejunum	(4.36 ± 1.47) × 10^10c^	(6.35 ± ± 0.46) × 10^9b^	0.003
Ileum	(4.81 ± 1.33) × 10^10c^	(7.74 ± 1.51) × 10^9b^	<0.001
Cecum	(2.13 ± 0.33) × 10^11bc^	(5.48 ± 0.29) × 10^9b^	0.012
Colon	(7.62 ± 1.64) × 10^11a^	(4.95 ± 0.42) × 10^9b^	0.047
Rectum	(7.35 ± 2.13) × 10^10c^	(4.04 ± 0.16) × 10^9b^	<0.001
*P* value§	<0.001	<0.001	

Note. Means within the same column with different subscripts are significantly different from one another.

^*^Copy number of 16S rRNA gene (copy.g^−1^).

^‡^Sampling site effect on bacterial density throughout the GIT of dairy cattle.

^§^Regional effect on bacterial density throughout the GIT of dairy calves.

## References

[b1] Savage D. C. Microbial ecology of the gastrointestinal tract. Annu Rev Microbiol 31, 107–133 (1977). 334036 10.1146/annurev.mi.31.100177.000543

[b2] Ley R. E. *et al.* Evolution of mammals and their gut microbes. Science 320, 1647–1651 (2008). 18497261 10.1126/science.1155725 PMC2649005

[b3] Sommer F. & Backhed F. The gut microbiota–masters of host development and physiology. Nat Rev Microbiol 11, 227–238 (2013). 23435359 10.1038/nrmicro2974

[b4] Mackie R. I. Mutualistic fermentative digestion in the gastrointestinal tract: diversity and evolution. Integr Comp Biol 42, 319–326 (2002). 21708724 10.1093/icb/42.2.319

[b5] de Oliveira M. N. *et al.* Characterizing the microbiota across the gastrointestinal tract of a Brazilian Nelore steer. Vet Microbiol 164, 307–314 (2013). 23490556 10.1016/j.vetmic.2013.02.013

[b6] Malmuthuge N., Griebel P. J. & Guan le L. Taxonomic identification of commensal bacteria associated with the mucosa and digesta throughout the gastrointestinal tracts of preweaned calves. Appl Environ Microbiol 80, 2021–2028 (2014). 24441166 10.1128/AEM.03864-13 PMC3957634

[b7] Smith M. I. *et al.* Gut microbiomes of Malawian twin pairs discordant for kwashiorkor. Science 339, 548–554 (2013). 23363771 10.1126/science.1229000 PMC3667500

[b8] Langille M. G. I. *et al.* Predictive functional profiling of microbial communities using 16S rRNA marker gene sequences. Nat Biotechnol 31, 814-+(2013). 23975157 10.1038/nbt.2676 PMC3819121

[b9] Reigstad C. S. & Kashyap P. C. Beyond phylotyping: understanding the impact of gut microbiota on host biology. Neurogastroenterol Motil 25, 358–372 (2013). 23594242 10.1111/nmo.12134 PMC4524550

[b10] Salonen A. & de Vos W. M. Impact of diet on human intestinal microbiota and health. Annu Rev Food Sci Technol 5, 239–262 (2014). 24387608 10.1146/annurev-food-030212-182554

[b11] Kim M., Morrison M. & Yu Z. T. Status of the phylogenetic diversity census of ruminal microbiomes. FEMS Microbiol Ecol 76, 49–63 (2011). 21223325 10.1111/j.1574-6941.2010.01029.x

[b12] Stevenson D. M. & Weimer P. J. Dominance of Prevotella and low abundance of classical ruminal bacterial species in the bovine rumen revealed by relative quantification real-time PCR. Appl Microbiol Biotechnol 75, 165–174 (2007). 17235560 10.1007/s00253-006-0802-y

[b13] Jami E. & Mizrahi I. Composition and Similarity of Bovine Rumen Microbiota across Individual Animals. Plos One 7, e33306 (2012). 22432013 10.1371/journal.pone.0033306 PMC3303817

[b14] Carroll I. M., Ringel-Kulka T., Siddle J. P. & Ringel Y. Alterations in composition and diversity of the intestinal microbiota in patients with diarrhea-predominant irritable bowel syndrome. Neurogastroenterol Motil 24, 521–530, e248 (2012). 22339879 10.1111/j.1365-2982.2012.01891.x PMC3975596

[b15] Malmuthuge N. *et al.* Distinct commensal bacteria associated with ingesta and mucosal epithelium in the gastrointestinal tracts of calves and chickens. FEMS Microbiol Ecol 79, 337–347 (2012). 22092450 10.1111/j.1574-6941.2011.01220.x

[b16] Collado M. C. & Sanz Y. Quantification of mucosa-adhered microbiota of lambs and calves by the use of culture methods and fluorescent *in situ* hybridization coupled with flow cytometry techniques. Vet Microbiol 121, 299–306 (2007). 17218070 10.1016/j.vetmic.2006.12.006

[b17] Marteyn B. *et al.* Modulation of Shigella virulence in response to available oxygen *in vivo*. Nature 465, 355–358 (2010). 20436458 10.1038/nature08970 PMC3750455

[b18] Pedron T. *et al.* A crypt-specific core microbiota resides in the mouse colon. MBio 3, 116–122 (2012). 10.1128/mBio.00116-12 PMC3372965 22617141

[b19] Baldwin RLt., Wu S., Li W., Li C., Bequette B. J. & Li R. W. Quantification of Transcriptome Responses of the Rumen Epithelium to Butyrate Infusion using RNA-seq Technology. Gene Regul Syst Bio 6, 67–80 (2012). 10.4137/GRSB.S9687 PMC3362330 22654504

[b20] Ferreira L. S. & Bittar C. M. Performance and plasma metabolites of dairy calves fed starter containing sodium butyrate, calcium propionate or sodium monensin. Animal 5, 239–245 (2011). 22440769 10.1017/S1751731110001965

[b21] Giacani L., Denisenko O., Tompa M. & Centurion-Lara A. Identification of the Treponema pallidum subsp. pallidum TP0092 (RpoE) regulon and its implications for pathogen persistence in the host and syphilis pathogenesis. J Bacter 195, 896–907 (2013). 10.1128/JB.01973-12 PMC3562100 23243302

[b22] Karlsson F., Klitgaard K. & Jensen T. K. Identification of Treponema pedis as the predominant Treponema species in porcine skin ulcers by fluorescence *in situ* hybridization and high-throughput sequencing. Vet Microbiol 171, 122–131 (2014). 24725449 10.1016/j.vetmic.2014.03.019

[b23] Naylor R. D., Martin P. K., Jones J. R. & Burnell M. C. Isolation of spirochaetes from an incident of severe virulent ovine footrot. Vet Rec 143, 690–691 (1998). 9921625

[b24] Stamm L. V., Walker R. L. & Read D. H. Genetic diversity of bovine ulcerative mammary dermatitis-associated Treponema. Vet Microbiol 136, 192–196 (2009). 19059737 10.1016/j.vetmic.2008.10.022

[b25] Walker R. L., Read D. H., Loretz K. J. & Nordhausen R. W. Spirochetes isolated from dairy cattle with papillomatous digital dermatitis and interdigital dermatitis. Vet Microbiol 47, 343–355 (1995). 8748549 10.1016/0378-1135(95)00114-x

[b26] Kim M. *et al.* Investigation of bacterial diversity in the feces of cattle fed different diets. J Anim Sci 92, 683–694 (2014). 24352967 10.2527/jas.2013-6841

[b27] Mao S., Zhang R., Wang D. & Zhu W. The diversity of the fecal bacterial community and its relationship with the concentration of volatile fatty acids in the feces during subacute rumen acidosis in dairy cows. BMC Vet Res 8, 237 (2012). 23217205 10.1186/1746-6148-8-237 PMC3582618

[b28] Nagy-Szakal D. *et al.* Maternal micronutrients can modify colonic mucosal microbiota maturation in murine offspring. Gut Microbes 3, 426–433 (2012). 22713270 10.4161/gmic.20697 PMC3679229

[b29] Erickson A. R. *et al.* Integrated metagenomics/metaproteomics reveals human host-microbiota signatures of Crohn’s disease. PLoS One 7, e49138 (2012). 23209564 10.1371/journal.pone.0049138 PMC3509130

[b30] Lamendella R., Domingo J. W., Ghosh S., Martinson J. & Oerther D. B. Comparative fecal metagenomics unveils unique functional capacity of the swine gut. BMC Microbiol 11, 103 (2011). 21575148 10.1186/1471-2180-11-103 PMC3123192

[b31] Arumugam M. *et al.* Enterotypes of the human gut microbiome. Nature 473, 174–180 (2011). 21508958 10.1038/nature09944 PMC3728647

[b32] Lu K. *et al.* Arsenic exposure perturbs the gut microbiome and its metabolic profile in mice: an integrated metagenomics and metabolomics analysis. Environ Health Perspect 122, 284–291 (2014). 24413286 10.1289/ehp.1307429 PMC3948040

[b33] Ridaura V. K. *et al.* Gut microbiota from twins discordant for obesity modulate metabolism in mice. Science 341, 1241214 (2013). 24009397 10.1126/science.1241214 PMC3829625

[b34] Caporaso J. G. *et al.* Ultra-high-throughput microbial community analysis on the Illumina HiSeq and MiSeq platforms. ISME J 6, 1621–1624 (2012). 22402401 10.1038/ismej.2012.8 PMC3400413

[b35] Campbell B. J., Polson S. W., Hanson T. E., Mack M. C. & Schuur E. A. The effect of nutrient deposition on bacterial communities in Arctic tundra soil. Environ Microbiol 12, 1842–1854 (2010). 20236166 10.1111/j.1462-2920.2010.02189.x

[b36] Edgar R. C. Search and clustering orders of magnitude faster than BLAST. Bioinformatics 26, 2460–2461 (2010). 20709691 10.1093/bioinformatics/btq461

[b37] DeSantis T. Z. *et al.* Greengenes, a chimera-checked 16S rRNA gene database and workbench compatible with ARB. Appl Environ Microbiol 72, 5069–5072 (2006). 16820507 10.1128/AEM.03006-05 PMC1489311

[b38] Caporaso J. G., Bittinger K., Bushman F. D., DeSantis T. Z., Andersen G. L. & Knight R. PyNAST: a flexible tool for aligning sequences to a template alignment. Bioinformatics 26, 266–267 (2010). 19914921 10.1093/bioinformatics/btp636 PMC2804299

[b39] Price M. N., Dehal P. S. & Arkin A. P. FastTree: computing large minimum evolution trees with profiles instead of a distance matrix. Mol Biol Evol 26, 1641–1650 (2009). 19377059 10.1093/molbev/msp077 PMC2693737

[b40] Wang Q., Garrity G. M., Tiedje J. M. & Cole J. R. Naive Bayesian classifier for rapid assignment of rRNA sequences into the new bacterial taxonomy. Appl Environ Microbiol 73, 5261–5267 (2007). 17586664 10.1128/AEM.00062-07 PMC1950982

[b41] Lozupone C. & Knight R. UniFrac: a new phylogenetic method for comparing microbial communities. Appl Environ Microbiol 71, 8228–8235 (2005). 16332807 10.1128/AEM.71.12.8228-8235.2005 PMC1317376

[b42] Schloss P. D. *et al.* Introducing mothur: open-source, platform-independent, community-supported software for describing and comparing microbial communities. Appl Environ Microbiol 75, 7537–7541 (2009). 19801464 10.1128/AEM.01541-09 PMC2786419

[b43] Kanehisa M. & Goto S. KEGG: kyoto encyclopedia of genes and genomes. Nucleic Acids Res 28, 27–30 (2000). 10592173 10.1093/nar/28.1.27 PMC102409

[b44] Hook S. E. *et al.* Impact of subacute ruminal acidosis (SARA) adaptation and recovery on the density and diversity of bacteria in the rumen of dairy cows. FEMS Microbiol Ecol 78, 275–284 (2011). 21692816 10.1111/j.1574-6941.2011.01154.x

[b45] Chen Y., Penner G. B., Li M., Oba M. & Guan L. L. Changes in bacterial diversity associated with epithelial tissue in the beef cow rumen during the transition to a high-grain diet. Appl Environ Microbiol 77, 5770–5781 (2011). 21705529 10.1128/AEM.00375-11 PMC3165274

[b46] Benjamini Y. & Hochberg Y. On the adaptive control of the false discovery rate in multiple testing with independent statistics. J Edu Behav Stats 25, 60–83 (2000).

