# Supplementary material for: Characterising the bacterial microbiota across the gastrointestinal tracts of dairy cattle: membership and potential function
Source: Sci Rep. 2015 Nov 3;5:16116. doi: 10.1038/srep16116 (PMC4630781; doi:10.1038/srep16116)
Supplement: Supplementary Information [file srep16116-s1.doc]

**Supplementary Figures and Tables**

**Characterising the bacterial microbiota across the gastrointestinal tracts of dairy cattle: membership and potential function**

Shengyong Mao*, Mengling Zhang, Junhua Liu, Weiyun Zhu

College of Animal Science and Technology, Nanjing Agricultural University, Nanjing 210095, China

**Supplementary Figure S1**

**Supplementary Figure S2**

**Supplementary Figure S3**

**Supplementary Figure S4**

**Supplementary Figure S5**

**Supplementary Figure S6**

**Supplementary Figure S7**

**Supplementary Table S1**

**Supplementary Table S2**

**Supplementary Table S3**

**Supplementary Table S4**

**Supplementary Table S5**

**Supplementary Table S6**

**Supplementary Table S7**

**Supplementary Table S8**

**Supplementary Table S9**

**Supplementary Table S10**

**Supplementary Table S11**

**Supplementary Table S12**

**Supplementary Table S13**

**Figure S1**. Summary of rarefaction results based on operational taxonomic unit (OTUs) (3% divergence) for each sample. A, Rarefaction curves are displayed for each of the luminal samples. B, Rarefaction curves are displayed for each of the mucosal samples. Rum-D-1, Rum-D-2, Rum-D-3, Rum-D-4, Rum-D-5, Rum-D-6, ruminal digesta samples; Ret-D-1, Ret-D-2, Ret-D-3, Ret-D-4, Ret-D-5, Ret-D-6, reticulal digesta samples; Oma-D-1, Oma-D-2, Oma-D-3, Oma-D-4, Oma-D-5, Oma-D-6, omasal digesta samples; Abo-D-1, Abo-D-2, Abo-D-3, Abo-D-4, Abo-D-5, Abo-D-6, abomasal digesta samples; Duo-D-1, Duo-D-2, Duo-D-3, Duo-D-4, Duo-D-5, Duo-D-6, duodenal digesta samples; Jej-D-1, Jej-D-2, Jej-D-3, Jej-D-4, Jej-D-5, Jej-D-6, jejumal digesta samples; Ile-D-1, Ile-D-2, Ile-D-3, Ile-D-4, Ile-D-5, Ile-D-6, ileal digesta samples; Cec-D-1, Cec-D-2, Cec-D-3, Cec-D-4, Cec-D-5, Cec-D-6, cecal digesta samples; Col-D-1, Col-D-2, Col-D-3, Col-D-4, Col-D-5, Col-D-6, colonal digesta samples; Rec-D-1, Rec-D-2, Rec-D-3, Rec-D-4, Rec-D-5, Rec-D-6, rectal digesta samples. Rum-M-1, Rum-M-2, Rum-M-3, Rum-M-4, Rum-M-5, Rum-M-6, ruminal mucosal samples; Ret-M-1, Ret-M-2, Ret-M-3, Ret-M-4, Ret-M-5, Ret-M-6, reticulal mucosal samples; Oma-M-1, Oma-M-2, Oma-M-3, Oma-M-4, Oma-M-5, Oma-M-6, omasal mucosal samples; Abo-M-1, Abo-M-2, Abo-M-3, Abo-M-4, Abo-M-5, Abo-M-6, abomasal mucosal samples; Duo-M-1, Duo-M-2, Duo-M-3, Duo-M-4, Duo-M-5, Duo-M-6, duodenal mucosal samples; Jej-M-1, Jej-M-2, Jej-M-3, Jej-M-4, Jej-M-5, Jej-M-6, jejumal mucosal samples; Ile-M-1, Ile-M-2, Ile-M-3, Ile-M-4, Ile-M-5, Ile-M-6, ileal mucosal samples; Cec-M-1, Cec-M-2, Cec-M-3, Cec-M-4, Cec-M-5, Cec-M-6, cecal mucosal samples; Col-M-1, Col-M-2, Col-M-3, Col-M-4, Col-M-5, Col-M-6, colonal mucosal samples; Rec-M-1, Rec-M-2, Rec-M-3, Rec-M-4, Rec-M-5, Rec-M-6, rectal mucosal samples.

**Figure S2**. The relative abundance of phylum across the gastrointestinal tract of dairy cattle.

**Figure S3**. Venn diagram of shared OTUs between the digesta and mucosa microbiomes.

**Figure S4.** Spatial distribution of most abundant OTUs (only the OTUs that average a relative abundance of ≥2% in at least one GIT region were presented) in the GITs of dairy cattle.

**Figure S5**. Comparisons of alpha diversity index and valid sequences detected between the digesta and its corresponding mucosal tissues across the gastrointestinal tract (GIT) of dairy cattle. A. The numbers of OTUs of each GIT site. B. The richness of each GIT site; C. The diversity of each GIT site. Boxes represent the interquartile range (IQR) between the first and third quartiles (25th and 75th percentiles, respectively) and the horizontal line inside the box defines the median. Whiskers represent the lowest and highest values within 1.5 times the IQR from the first and third quartiles, respectively. Boxes with the star symbol above their whiskers are significantly different between the digesta and its corresponding mucosa in each sites of GIT at *P* < 0.05 using t-test analysis.

**Figure S6.** Comparison of the relative abundance of predominant genera (relative abundance ≥5% in at least one gastrointestinal tract region) between the digesta and its corresponding mucosal samples across the gastrointestinal tract of dairy cattle. Error bars represent a single standard deviation. The star symbol indicates that there is a significantly different between the digesta and its corresponding mucosal samples (*P* < 0.05).

**Figure S7.** Comparison of the gene counts for selected functional individual pathways for samples from digesta and mucosal samples across the gastrointestinal tract(GIT) of dairy cattle. Error bars represent a single standard deviation. The individual pathways shown include the following KEGG categories: (A) membrane transport; (B) replication and repair; (C) carbohydrate metabolism; (D) amino acid metabolism; (E) energy metabolism. The star symbol indicates that are significantly different (*P* < 0.05).

**Table S1**. Number of sequences, estimated sample coverage, diversity and OTU richness in each sample.

| Sampling type | Region | Sample ID | Valid sequences | OTUs | Chao | Shannon | Coverage |
| --- | --- | --- | --- | --- | --- | --- | --- |
| Digesta |  |  |  |  |  |  |  |
|  | Rumen | Rum-D-1 | 48296 | 2565 | 3118 | 6.39 | 0.986 |
|  |  | Rum-D-2 | 58547 | 2534 | 3207 | 6.24 | 0.989 |
|  |  | Rum-D-3 | 44106 | 2347 | 2935 | 6.29 | 0.986 |
|  |  | Rum-D-4 | 41834 | 2200 | 2891 | 6.12 | 0.985 |
|  |  | Rum-D-5 | 52346 | 2397 | 3109 | 6.24 | 0.988 |
|  |  | Rum-D-6 | 41416 | 2298 | 3043 | 6.28 | 0.984 |
|  | Reticulum | Ret-D-1 | 46462 | 2282 | 2649 | 6.32 | 0.99 |
|  |  | Ret-D-2 | 53358 | 2267 | 2718 | 6.25 | 0.991 |
|  |  | Ret-D-3 | 44134 | 1986 | 2411 | 6.13 | 0.989 |
|  |  | Ret-D-4 | 35293 | 1889 | 2266 | 6.15 | 0.987 |
|  |  | Ret-D-5 | 51339 | 2162 | 2663 | 6.15 | 0.99 |
|  |  | Ret-D-6 | 42949 | 2068 | 2520 | 6.14 | 0.989 |
|  | Omasum | Oma-D-1 | 40595 | 2044 | 2417 | 6.09 | 0.988 |
|  |  | Oma-D-2 | 36362 | 1905 | 2339 | 6.03 | 0.987 |
|  |  | Oma-D-3 | 46147 | 2055 | 2570 | 6.06 | 0.989 |
|  |  | Oma-D-4 | 40005 | 1946 | 2433 | 6.11 | 0.988 |
|  |  | Oma-D-5 | 54949 | 2134 | 2563 | 6.15 | 0.991 |
|  |  | Oma-D-6 | 47914 | 2072 | 2530 | 6.05 | 0.99 |
|  | Abomasum | Abo-D-1 | 41459 | 2196 | 2632 | 6.29 | 0.988 |
|  |  | Abo-D-2 | 55320 | 1876 | 2431 | 5.54 | 0.991 |
|  |  | Abo-D-3 | 44075 | 2000 | 2527 | 5.8 | 0.988 |
|  |  | Abo-D-4 | 39045 | 2044 | 2515 | 6.07 | 0.987 |
|  |  | Abo-D-5 | 45316 | 2534 | 3249 | 6.2 | 0.984 |
|  |  | Abo-D-6 | 56806 | 2290 | 2683 | 6.21 | 0.991 |
|  | Duodenum | Duo-D-1 | 52116 | 1426 | 2061 | 3.44 | 0.99 |
|  |  | Duo-D-2 | 46694 | 1105 | 1713 | 3.25 | 0.991 |
|  |  | Duo-D-3 | 41088 | 1048 | 1650 | 3.59 | 0.989 |
|  |  | Duo-D-4 | 34831 | 1171 | 1738 | 3.46 | 0.987 |
|  |  | Duo-D-5 | 37431 | 1249 | 1915 | 3.31 | 0.987 |
|  |  | Duo-D-6 | 36045 | 1228 | 1842 | 3.89 | 0.987 |
|  | Jejunum | Jej-D-1 | 50858 | 1360 | 2157 | 3.98 | 0.989 |
|  |  | Jej-D-2 | 31105 | 1219 | 1973 | 4.44 | 0.983 |
|  |  | Jej-D-3 | 44721 | 1500 | 2086 | 5.18 | 0.989 |
|  |  | Jej-D-4 | 49964 | 1385 | 1938 | 4.89 | 0.991 |
|  |  | Jej-D-5 | 35136 | 1354 | 2067 | 4.94 | 0.985 |
|  |  | Jej-D-6 | 34050 | 1377 | 1838 | 5.29 | 0.987 |
|  | Ileum | Ile-D-1 | 49807 | 1148 | 1677 | 3.52 | 0.991 |
|  |  | Ile-D-2 | 57664 | 1076 | 1726 | 3.26 | 0.992 |
|  |  | Ile-D-3 | 41455 | 899 | 1349 | 3.34 | 0.992 |
|  |  | Ile-D-4 | 49691 | 834 | 1292 | 3.01 | 0.993 |
|  |  | Ile-D-5 | 37776 | 1457 | 2419 | 4.08 | 0.983 |
|  |  | Ile-D-6 | 54032 | 1127 | 1546 | 3.79 | 0.993 |
|  | Cecum | Cec-D-1 | 33917 | 1078 | 1675 | 3.65 | 0.988 |
|  |  | Cec-D-2 | 59475 | 1082 | 1539 | 3.12 | 0.994 |
|  |  | Cec-D-3 | 50252 | 918 | 1335 | 3.07 | 0.993 |
|  |  | Cec-D-4 | 52042 | 912 | 1272 | 3.05 | 0.994 |
|  |  | Cec-D-5 | 54291 | 823 | 1228 | 2.85 | 0.994 |
|  |  | Cec-D-6 | 46419 | 889 | 1221 | 3.02 | 0.993 |
|  | Colon | Col-D-1 | 50842 | 1350 | 1686 | 4.13 | 0.993 |
|  |  | Col-D-2 | 47662 | 911 | 1312 | 2.87 | 0.993 |
|  |  | Col-D-3 | 45281 | 782 | 1195 | 3.03 | 0.993 |
|  |  | Col-D-4 | 53305 | 928 | 1334 | 2.99 | 0.993 |
|  |  | Col-D-5 | 61132 | 1042 | 1468 | 2.99 | 0.994 |
|  |  | Col-D-6 | 47867 | 1118 | 1589 | 3.45 | 0.992 |
|  | Rectum | Rec-D-1 | 40509 | 1351 | 1793 | 4.45 | 0.99 |
|  |  | Rec-D-2 | 35690 | 1005 | 1488 | 3.48 | 0.989 |
|  |  | Rec-D-3 | 61251 | 1061 | 1489 | 3.16 | 0.994 |
|  |  | Rec-D-4 | 37750 | 842 | 1275 | 3.08 | 0.991 |
|  |  | Rec-D-5 | 51788 | 968 | 1289 | 3.37 | 0.994 |
|  |  | Rec-D-6 | 59646 | 1290 | 1823 | 3.4 | 0.992 |
| Mucosa tissue | |  |  |  |  |  |  |
|  | Rumen | Rum-M-1 | 41474 | 1790 | 2459 | 5.31 | 0.985 |
|  |  | Rum-M-2 | 53567 | 2126 | 2685 | 5.71 | 0.989 |
|  |  | Rum-M-3 | 58237 | 1894 | 2582 | 5.21 | 0.989 |
|  |  | Rum-M-4 | 64464 | 2407 | 3116 | 5.82 | 0.99 |
|  |  | Rum-M-5 | 39311 | 1969 | 2687 | 5.69 | 0.984 |
|  |  | Rum-M-6 | 45716 | 1673 | 2242 | 5.05 | 0.988 |
|  | Reticulum | Ret-M-1 | 56004 | 2316 | 2913 | 5.79 | 0.989 |
|  |  | Ret-M-2 | 64480 | 2317 | 2811 | 5.96 | 0.992 |
|  |  | Ret-M-3 | 42226 | 1955 | 2528 | 5.76 | 0.986 |
|  |  | Ret-M-4 | 61040 | 2258 | 2837 | 5.88 | 0.99 |
|  |  | Ret-M-5 | 42992 | 2328 | 2971 | 6.18 | 0.985 |
|  |  | Ret-M-6 | 49441 | 2024 | 2617 | 5.56 | 0.988 |
|  | Omasum | Oma-M-1 | 38413 | 2323 | 2871 | 6.15 | 0.984 |
|  |  | Oma-M-2 | 41917 | 2438 | 2936 | 6.3 | 0.986 |
|  |  | Oma-M-3 | 58731 | 2512 | 3037 | 6.39 | 0.99 |
|  |  | Oma-M-4 | 39215 | 2270 | 2798 | 5.99 | 0.985 |
|  |  | Oma-M-5 | 38046 | 2340 | 2931 | 6.32 | 0.984 |
|  |  | Oma-M-6 | 52597 | 2447 | 2913 | 6.13 | 0.99 |
|  | Abomasum | Abo-M-1 | 67866 | 2404 | 2779 | 5.64 | 0.993 |
|  |  | Abo-M-2 | 48035 | 1902 | 2325 | 4.49 | 0.991 |
|  |  | Abo-M-3 | 57524 | 2089 | 2544 | 4.32 | 0.991 |
|  |  | Abo-M-4 | 52138 | 2545 | 3046 | 6.21 | 0.989 |
|  |  | Abo-M-5 | 63990 | 2547 | 2980 | 5.64 | 0.992 |
|  |  | Abo-M-6 | 57826 | 2397 | 2714 | 5.99 | 0.993 |
|  | Duodenum | Duo-M-1 | 65555 | 1186 | 1712 | 5.41 | 0.994 |
|  |  | Duo-M-2 | 59444 | 1195 | 1788 | 5.08 | 0.993 |
|  |  | Duo-M-3 | 63448 | 1302 | 1926 | 5.32 | 0.993 |
|  |  | Duo-M-4 | 74103 | 1519 | 2231 | 5.72 | 0.994 |
|  |  | Duo-M-5 | 71430 | 1308 | 2028 | 5.34 | 0.994 |
|  |  | Duo-M-6 | 53548 | 1248 | 1854 | 5.26 | 0.992 |
|  |  |  |  |  |  |  |  |
|  | Jejunum | Jej-M-1 | 59159 | 1563 | 2504 | 5.32 | 0.989 |
|  |  | Jej-M-2 | 57755 | 1265 | 2005 | 5.23 | 0.991 |
|  |  | Jej-M-3 | 76036 | 1865 | 2720 | 5.46 | 0.991 |
|  |  | Jej-M-4 | 9930 | 714 | 1201 | 5.19 | 0.972 |
|  |  | Jej-M-5 | 49113 | 1270 | 2060 | 4.87 | 0.989 |
|  |  | Jej-M-6 | 65092 | 1655 | 2465 | 5.54 | 0.99 |
|  | Ileum | Ile-M-1 | 32583 | 1144 | 1605 | 5.6 | 0.991 |
|  |  | Ile-M-2 | 52239 | 1360 | 2127 | 5.2 | 0.99 |
|  |  | Ile-M-3 | 66367 | 1321 | 2138 | 5.06 | 0.992 |
|  |  | Ile-M-4 | 50098 | 1369 | 2002 | 4.88 | 0.991 |
|  |  | Ile-M-5 | 57684 | 1096 | 1764 | 4.67 | 0.992 |
|  |  | Ile-M-6 | 62000 | 1480 | 2256 | 5.3 | 0.991 |
|  | Cecum | Cec-M-1 | 55838 | 1800 | 2414 | 5.51 | 0.991 |
|  |  | Cec-M-2 | 53370 | 1565 | 2065 | 4.97 | 0.992 |
|  |  | Cec-M-3 | 35633 | 989 | 1590 | 4.97 | 0.99 |
|  |  | Cec-M-4 | 54957 | 1561 | 2329 | 4.9 | 0.991 |
|  |  | Cec-M-5 | 65515 | 1620 | 2273 | 4.39 | 0.992 |
|  |  | Cec-M-6 | 61484 | 1685 | 2372 | 5.66 | 0.992 |
|  | Colon | Col-M-1 | 42397 | 1709 | 2494 | 6.09 | 0.987 |
|  |  | Col-M-2 | 74290 | 1952 | 2540 | 5.43 | 0.993 |
|  |  | Col-M-3 | 55467 | 1545 | 2068 | 4.95 | 0.992 |
|  |  | Col-M-4 | 58424 | 1401 | 1962 | 5.11 | 0.993 |
|  |  | Col-M-5 | 48258 | 1603 | 2149 | 5.3 | 0.991 |
|  |  | Col-M-6 | 52968 | 1488 | 2199 | 5.49 | 0.991 |
|  | Rectum | Rec-M-1 | 64790 | 2750 | 3127 | 6.26 | 0.992 |
|  |  | Rec-M-2 | 48284 | 1791 | 2311 | 5.85 | 0.99 |
|  |  | Rec-M-3 | 63891 | 1562 | 2158 | 5.44 | 0.994 |
|  |  | Rec-M-4 | 48746 | 1872 | 2296 | 5.74 | 0.991 |
|  |  | Rec-M-5 | 48828 | 1577 | 2010 | 4.99 | 0.991 |
|  |  | Rec-M-6 | 65552 | 1521 | 2179 | 5.5 | 0.993 |

**Table S2**. Analysis of molecular variation hypothesis testing results in determining the difference in structure of the bacterial population of digesta and mucosal tissues across the gastrointestinal tract of dairy cattle. The star symbol indicated there is a significant difference in the structure of the bacterial population between the two sampling sites.

|  | Rumen | Reticulum | Omasum | Abomasum | Duodenum | Jejunum | Ileum | Cecum | Colon | Rectum |
| --- | --- | --- | --- | --- | --- | --- | --- | --- | --- | --- |
| **Digesta** |  |  |  |  |  |  |  |  |  |  |
| Rumen |  | 0.001* | 0.001^*^ | 0.002* | <0.001* | 0.002* | 0.001* | 0.001* | 0.007* | 0.002* |
| Reticulum | 0.001* |  | <0.001* | 0.001* | 0.001* | 0.003* | 0.004* | 0.003* | 0.004* | 0.002* |
| Omasum | 0.001* | <0.001* |  | 0.002* | <0.001* | 0.001* | 0.003* | 0.004* | 0.002* | 0.002* |
| Abomasum | 0.002* | 0.001* | 0.002* |  | 0.001* | 0.002* | 0.003* | 0.002* | 0.003* | 0.002* |
| Duodenum | <0.001* | 0.001* | <0.001* | 0.001* |  | 0.123 | 0.001* | 0.003* | 0.004* | 0.001* |
| Jejunum | 0.002* | 0.003* | 0.001* | 0.002* | 0.123 |  | <0.001* | 0.002* | 0.003* | 0.006* |
| Ileum | 0.001* | 0.004* | 0.003* | 0.003* | 0.001* | <0.001* |  | 0.005* | 0.001* | 0.002* |
| Cecum | 0.001* | 0.003* | 0.004* | 0.002* | 0.003* | 0.002* | 0.005* |  | 0.179 | 0.052 |
| Colon | 0.007* | 0.004* | 0.002* | 0.003* | 0.004* | 0.003* | 0.001* | 0.179 |  | 0.485 |
| Rectum | 0.002* | 0.002* | 0.002* | 0.002* | 0.001* | 0.006* | 0.002* | 0.052 | 0.485 |  |
| **Mucosa** |  |  |  |  |  |  |  |  |  |  |
| Rumen |  | 0.027* | 0.002* | 0.001* | 0.003* | <0.001* | 0.004* | 0.006* | 0.002* | 0.001* |
| Reticulum | 0.027* |  | <0.001* | <0.001* | 0.001* | <0.001* | <0.001* | 0.001* | 0.001* | 0.001* |
| Omasum | 0.002* | <0.001* |  | 0.082 | 0.001* | 0.001* | 0.002* | 0.002* | 0.001* | 0.002* |
| Abomasum | 0.001* | <0.001* | 0.082 |  | 0.002* | 0.001* | 0.003* | <0.001* | 0.001* | 0.004* |
| Duodenum | 0.003* | 0.001* | 0.001* | 0.002* |  | 0.002* | 0.002* | 0.002* | 0.003 | 0.003* |
| Jejunum | 0.001* | <0.001* | 0.001* | 0.001* | 0.002* |  | 0.328 | 0.037 | 0.015* | 0.001* |
| Ileum | 0.004* | <0.001* | 0.002* | 0.003* | 0.002* | 0.328 |  | 0.016 | 0.001* | 0.001* |
| Cecum | 0.006* | 0.001* | 0.002* | <0.001* | 0.002* | 0.037* | 0.016* |  | 0.949 | 0.224 |
| Colon | 0.002* | 0.001* | 0.001* | 0.001* | 0.003* | 0.015* | 0.001* | 0.949 |  | 0.585 |
| Rectum | <0.001* | 0.001* | 0.002* | 0.004* | 0.003* | 0.001* | 0.001* | 0.224 | 0.585 |  |

**Table S3**. Comparison of the phyla in digesta samples across the gastrointestinal tract of dairy cattle (the multiple comparisons results were only presented for the phyla which average relative abundance ≥1% in at least one region). Mean in the same row with different superscripts represents a significant difference (*P* < 0.05)

| Phylum | Rumen | Reticulum | Omasum | Abomasum | Duodenum | Jejunum | Ileum | Cecum | Colon | Rectum | SEM | P value | FDR^*^ |
| --- | --- | --- | --- | --- | --- | --- | --- | --- | --- | --- | --- | --- | --- |
| Firmicutes | 50.49^ef^ | 53.86^de^ | 50.96^ef^ | 64.37^cd^ | 38.28^f^ | 68.52^bc^ | 67.49^bc^ | 79.15^ab^ | 83.71^a^ | 91.26^a^ | 2.211 | <0.001 | <0.001 |
| Bacteroidetes | 42.39^a^ | 38.98^a^ | 40.59^a^ | 19.66^b^ | 1.78^c^ | 1.66^c^ | 1.31^c^ | 0.41^c^ | 0.99^c^ | 2.87^c^ | 2.353 | <0.001 | <0.001 |
| Proteobacteria | 1.15^e^ | 1.12^e^ | 1.21^e^ | 3.48^de^ | 53.87^a^ | 17.49^bc^ | 26.86^b^ | 15.65^bcd^ | 10.62^cde^ | 1.4^e^ | 2.223 | <0.001 | <0.001 |
| Actinobacteria | 1.27^b^ | 1.23^b^ | 1.13^b^ | 5.18^b^ | 4.29^b^ | 9.62^a^ | 3.12^b^ | 3.97^b^ | 3.32^b^ | 2.96^b^ | 0.402 | <0.001 | <0.001 |
| Tenericutes | 1.43^b^ | 1.43^b^ | 2.11^a^ | 1.03^b^ | 0.2^c^ | 0.23^c^ | 0.24^c^ | 0.1^c^ | 0.18^c^ | 0.35^c^ | 0.096 | <0.001 | <0.001 |
| Spirochaetae | 1.02^b^ | 0.91^bc^ | 0.59^bcd^ | 2.03^a^ | 0.06^d^ | 0.07^d^ | 0.04^d^ | 0.08^d^ | 0.31^cd^ | 0.27^cd^ | 0.089 | <0.001 | <0.001 |
| Lentisphaerae | 0.35^bc^ | 0.3^bc^ | 0.5^b^ | 1.12^a^ | 0.05^bc^ | 0.14^bc^ | 0.05^bc^ | 0.01^bc^ | 0.01^bc^ | 0.01^c^ | 0.053 | <0.001 | <0.001 |
| Unclassified Bacteria | 1.6b^cde^ | 1.67^abcd^ | 2.57^a^ | 2.23^ab^ | 1.23^cdef^ | 1.97^abc^ | 0.78^def^ | 0.59^f^ | 0.76^def^ | 0.68^ef^ | 0.105 | <0.001 | <0.001 |
| Chloroflexi | 0.13 | 0.1 | 0.06 | 0.05 | 0.03 | 0.02 | 0.02 | 0.005 | 0.004 | 0.003 | 0.009 | 0.004 | 0.005 |
| Acidobacteria | <0.001 | ND | ND | ND | ND | ND | ND | ND | ND | ND | <0.001 | <0.001 | <0.001 |
| Armatimonadetes | 0.01 | 0.01 | 0.02 | 0.02 | 0.002 | 0.001 | ND | ND | 0.003 | ND | 0.001 | <0.001 | <0.001 |
| Chlamydiae | <0.001 | ND | ND | ND | ND | ND | <0.001 | ND | ND | ND | 0.000 | 0.537 | 0.537 |
| Chlorobi | ND | ND | ND | 0.01 | 0.01 | ND | ND | ND | ND | ND | 0.001 | 0.524 | 0.537 |
| Cyanobacteria | 0.07 | 0.26 | 0.17 | 0.25 | 0.09 | 0.19 | 0.04 | 0.03 | 0.04 | 0.06 | 0.021 | 0.038 | 0.04 |
| Deinococcus-Thermus | 0.002 | ND | <0.001 | 0.001 | ND | ND | <0.001 | ND | <0.00 | ND | 0.000 | 0.003 | 0.004 |
| Elusimicrobia | 0.02 | 0.01 | 0.04 | 0.09 | 0.003 | 0.003 | 0.001 | ND | ND | <0.001 | 0.005 | <0.001 | <0.001 |
| Fibrobacteres | 0.02 | 0.08 | 0.02 | 0.35 | 0.01 | 0.01 | 0.002 | ND | <0.001 | 0.003 | 0.019 | <0.001 | <0.001 |
| Fusobacteria | 0.02 | 0.02 | 0.01 | 0.06 | 0.06 | 0.06 | 0.02 | 0.005 | 0.005 | 0.01 | 0.006 | 0.017 | 0.021 |
| Planctomycetes | ND | ND | <0.001 | 0.03 | 0.02 | ND | ND | ND | ND | ND | 0.003 | 0.526 | 0.537 |
| Synergistetes | 0.02 | 0.01 | 0.01 | 0.04 | 0.001 | 0.003 | 0.003 | ND | ND | <0.001 | 0.002 | <0.001 | <0.001 |
| Verrucomicrobia | 0.02 | 0.01 | <0.001 | 0.01 | 0.002 | 0.003 | 0.01 | 0.02 | 0.05 | 0.13 | 0.007 | <0.001 | <0.001 |

ND, not detected

^*^FDR: False discovery rate.

**Table S4.** Comparison of the predominant genera (average relative abundance ≥5% in at least one GIT region) in digesta samples across the gastrointestinal tract of dairy cattle. Mean in the same row with different superscripts represents a significant difference (*P* < 0.05).

| Taxa | Rumen | Reticulum | Omasum | Abomasum | Duodenum | Jejunum | Ileum | Cecum | Colon | Rectum | SEM | P value | FDR^*^ |
| --- | --- | --- | --- | --- | --- | --- | --- | --- | --- | --- | --- | --- | --- |
| Bacteroidetes |  |  |  |  |  |  |  |  |  |  |  |  |  |
| *Prevotella* | 19.08^a^ | 16.01^a^ | 10.57^b^ | 5.89^c^ | 0.57^d^ | 0.5^d^ | 0.06^d^ | 0.01^d^ | 0.01^d^ | 0.04^d^ | 0.954 | < 0.001 | < 0.001 |
| Unclassified Rikenellaceae | 9.65^ab^ | 8.59b | 11.14^a^ | 4.28^c^ | 0.51^d^ | 0.45^d^ | 0.14^d^ | 0.12^d^ | 0.32^d^ | 1.16^d^ | 0.571 | < 0.001 | < 0.001 |
| Unclassified Bacteroidales | 8.58^bc^ | 10.29^ab^ | 13.2^a^ | 5.78^c^ | 0.45^d^ | 0.45^d^ | 0.11^d^ | 0.08^d^ | 0.18^d^ | 0.57^d^ | 0.653 | < 0.001 | < 0.001 |
| Firmicutes |  |  |  |  |  |  |  |  |  |  |  |  |  |
| Unclassified Ruminococcaceae | 13.59^ab^ | 14.89^a^ | 16.11^a^ | 16.16^a^ | 2.41^c^ | 4.31^c^ | 2.03^c^ | 3.85^c^ | 5.31^c^ | 8.27^bc^ | 0.831 | < 0.001 | < 0.001 |
| Unclassified Christensenellaceae | 8.73^a^ | 8.78^a^ | 6.76^ab^ | 5.78^b^ | 2.37^cd^ | 4.81^bc^ | 1.11^d^ | 1.25^d^ | 1.68^d^ | 2.25^d^ | 0.412 | < 0.001 | < 0.001 |
| Unclassified Lachnospiraceae | 6.15^b^ | 6.54^b^ | 5.77^c^ | 6.69^b^ | 6.15^b^ | 9.29^a^ | 2.75^d^ | 1.21^de^ | 1^e^ | 0.77^e^ | 0.383 | < 0.001 | < 0.001 |
| *Butyrivibrio* | 3.75^d^ | 4.33^cd^ | 4.3^cd^ | 7.95^bc^ | 9.24^b^ | 11.53^ab^ | 3.79^d^ | 2.52^d^ | 2.02^d^ | 1.55^d^ | 0.482 | < 0.001 | < 0.001 |
| *Ruminococcus* | 2.44^cd^ | 3.16^cd^ | 3.3^c^ | 5.42^b^ | 3.19^c^ | 7.42^a^ | 2.63^cd^ | 1.94^cd^ | 1.95^cd^ | 1.49^d^ | 0.252 | < 0.001 | < 0.001 |
| *Acetitomaculum* | 2.24cde | 2.11^de^ | 1.8^de^ | 4.52^b^ | 4.26^bc^ | 7.91^a^ | 3.13^bcd^ | 1.26^de^ | 1.1^de^ | 0.55^e^ | 0.300 | < 0.001 | < 0.001 |
| Unclassified Clostridiales | 1.88e | 2.39^de^ | 1.96^e^ | 4.15^bc^ | 3.6^cd^ | 5.31^ab^ | 1.59^e^ | 1.29^e^ | 1.19^e^ | 1.2^7e^ | 0.191 | < 0.001 | < 0.001 |
| Unclassified Peptostreptococcaceae | 0.42e | 0.14^e^ | 0.16^e^ | 0.16^e^ | 0.5^e^ | 6.09^e^ | 33.2^d^ | 45.44^abc^ | 42.56^bcd^ | 41.29^cd^ | 2.632 | < 0.001 | < 0.001 |
| *Turicibacter* | 0.14b | 0.03^b^ | 0.03^b^ | 0.04^b^ | 0.11^b^ | 1.3^b^ | 10.91^a^ | 12.03^a^ | 13.19^a^ | 14.23^a^ | 0.841 | < 0.001 | < 0.001 |
| *Clostridium* | 0.11d | 0.02^d^ | 0.02^d^ | 0.08^d^ | 0.08^d^ | 0.09^d^ | 1.75^d^ | 5.64^c^ | 9.81^b^ | 14.72^a^ | 0.682 | < 0.001 | < 0.001 |
| Proteobacteria |  |  |  |  |  |  |  |  |  |  |  |  |  |
| Unclassified Enterobacteriaceae | 0.2c | 0.67^c^ | 0.72^c^ | 1.03^c^ | 46.3^a^ | 13.97^b^ | 21.5^b^ | 15.29^b^ | 10.34^bc^ | 1.13^c^ | 1.953 | < 0.001 | < 0.001 |

^*^FDR: False discovery rate.

**Table S5**. Comparison of the dominant operational taxonomic units (OTU) (relative abundance ≥2% in at least one region of gastrointestinal tract(GIT)) in digesta samples across the GIT of dairy cattle. Means in the same row with different superscripts represents a significant difference (*P* < 0.05).

| No. OTU ID^2^ | Classification | Percentage of total sequences ^1^ | | | | | | | | | | SEM | P value | FDR* |
| --- | --- | --- | --- | --- | --- | --- | --- | --- | --- | --- | --- | --- | --- | --- |
|  |  | Rumen-D | Reticulum-D | Omasum-D | Abomasum-D | Duodenum-D | Jejunum-D | Ileum-D | Cecum-D | Colon-D | Rectum-D |  |  |  |
| OTU5930 | *Acetitomaculum* | 0.76^bcde^ | 0.71^cde^ | 0.64^cde^ | 1.68^bc^ | 1.86^b^ | 3.59^a^ | 1.62^bcd^ | 0.56^de^ | 0.45^e^ | 0.14^e^ | 0.144 | <0.001 | <0.001 |
| OTU2613 | *Bifidobacterium* | 0.02^b^ | 0.02^b^ | 0.03^b^ | 0.08^b^ | 0.06^b^ | 0.8^ab^ | 1.13^ab^ | 2.64^a^ | 2.11^ab^ | 2.12^ab^ | 0.198 | <0.001 | <0.001 |
| OTU3071 | *Butyrivibrio* | 0.5^bc^ | 0.73^bc^ | 0.67^bc^ | 1.34^ab^ | 2.1^a^ | 2.28^a^ | 0.7^bc^ | 0.34^bc^ | 0.24^c^ | 0.2^c^ | 0.111 | <0.001 | <0.001 |
| OTU369 | *Butyrivibrio* | 0.94^c^ | 0.85^c^ | 0.9^c^ | 2.^5ab^ | 1.7^c^ | 3.08^a^ | 1.09^c^ | 1.08^c^ | 0.97^c^ | 0.75^c^ | 0.119 | <0.001 | <0.001 |
| OTU604 | *Clostridium* | 0.1d | 0.02^d^ | 0.02^d^ | 0.03^d^ | 0.06^d^ | 0.07^d^ | 1.62^d^ | 5.14^c^ | 9.07^b^ | 13.48^a^ | 0.623 | <0.001 | <0.001 |
| OTU4038 | *Mogibacterium* | 0.68^bc^ | 0.92^bc^ | 0.91^bc^ | 1.81^ab^ | 2.31^a^ | 2.46a | 0.98^bc^ | 0.42^c^ | 0.66^bc^ | 0.46^c^ | 0.118 | <0.001 | <0.001 |
| OTU1131 | *Pseudobutyrivibrio* | 0.1^b^ | 0.24^b^ | 0.41^b^ | 2.1^a^ | 0.17^b^ | 0.69b | 0.47^b^ | 0.03^b^ | 0.02^b^ | 0.01^b^ | 0.093 | <0.001 | <0.001 |
| OTU654 | *Pseudomonas* | 0.02^c^ | 0.04^c^ | 0.04^c^ | 0.14^c^ | 2.03^a^ | 1.07^b^ | 0.02^c^ | 0.01^c^ | 0.01^c^ | 0.01^c^ | 0.088 | <0.001 | <0.001 |
| OTU6273 | *Ruminococcus* | 0.5^b^ | 0.72^b^ | 0.74^b^ | 1.16^b^ | 0.88^b^ | 2.39^a^ | 0.94^b^ | 0.73^b^ | 0.77^b^ | 0.66^b^ | 0.092 | <0.001 | <0.001 |
| OTU1315 | *Turicibacter* | 0.12^b^ | 0.02^b^ | 0.02^b^ | 0.04^b^ | 0.09^b^ | 1.15^b^ | 9.64^a^ | 10.31^a^ | 11.4^a^ | 12.26^a^ | 0.726 | <0.001 | <0.001 |
| OTU583 | Unclassified Christensenellaceae | 2.89^a^ | 2.69^a^ | 1.65^b^ | 1.18^bc^ | 0.41^d^ | 0.79^cd^ | 0.18^d^ | 0.08^d^ | 0.09^d^ | 0.07^d^ | 0.141 | <0.001 | <0.001 |
| OTU3825 | Unclassified Enterobacteriaceae | 0.19^c^ | 0.65^c^ | 0.7^c^ | 1^c^ | 45.32^a^ | 13.5^b^ | 21.12^b^ | 15.03^b^ | 10.18^bc^ | 1.1^c^ | 1.906 | <0.001 | <0.001 |
| OTU3338 | Unclassified Lachnospiraceae | 0.5^de^ | 0.42^de^ | 0.36^de^ | 0.59^c^ | 1.42^b^ | 2.02^a^ | 0.51^de^ | 0.21^de^ | 0.15^de^ | 0.08^e^ | 0.081 | <0.001 | <0.001 |
| OTU2405 | Unclassified Peptostreptococcaceae | 0.03^b^ | 0b | 0.01^b^ | 0.01^b^ | 0.01^b^ | 0.29^b^ | 1.7^a^ | 2.12^a^ | 2.44^a^ | 2.12^a^ | 0.143 | <0.001 | <0.001 |
| OTU4730 | Unclassified Peptostreptococcaceae | 0.2^c^ | 0.07^c^ | 0.07^c^ | 0.07^c^ | 0.25^c^ | 2.5^c^ | 15.58^b^ | 22.96^a^ | 21.64^a^ | 20.53^a^ | 1.312 | <0.001 | <0.001 |
| OTU4916 | Unclassified Peptostreptococcaceae | 0.17^b^ | 0.06^b^ | 0.07^b^ | 0.07^b^ | 0.19^b^ | 2.95^b^ | 13.62^a^ | 16.12^a^ | 14.97^a^ | 14.44^a^ | 0.982 | <0.001 | <0.001 |
| OTU3160 | Unclassified Rikenellaceae | 2.97^b^ | 2.65^b^ | 4.65^a^ | 1.58^c^ | 0.19^d^ | 0.16^d^ | 0.01^d^ | <0.001^d^ | <0.001^d^ | <0.001^d^ | 0.215 | <0.001 | <0.001 |
| OTU120 | Unclassified Ruminococcaceae | 3.51^a^ | 3.66^a^ | 3.81^a^ | 2.85^a^ | 0.4^b^ | 0.53^b^ | 0.14^b^ | 0.04^b^ | 0.03^b^ | 0.04^b^ | 0.225 | <0.001 | <0.001 |

^1^Values represent means.

^2^A total of 6860 OTUs were numbered in serial order.

**Table S6**. Comparison of the phyla in mucosal samples across the gastrointestinal tract of dairy cattle(the multiple comparisons results were only presented for the phyla which average relative abundance ≥1% in at least one region). Mean in the same row with different superscripts represents a significant difference (*P* < 0.05).

| Phylum | Rumen | Reticulum | Omasum | Abomasum | Duodenum | Jejunum | Ileum | Cecum | Colon | Rectum | SEM | P value | FDR^*^ |
| --- | --- | --- | --- | --- | --- | --- | --- | --- | --- | --- | --- | --- | --- |
| Firmicutes | 43.79^ab^ | 43.01^ab^ | 39.43^ab^ | 27.4^b^ | 34.21^ab^ | 42.24^ab^ | 52.15^a^ | 48.29^ab^ | 45.17^ab^ | 46.57^ab^ | 1.614 | 0.026 | 0.047 |
| Bacteroidetes | 21.98^bc^ | 26.95^ab^ | 39.08a | 20.95^bc^ | 10.33^cd^ | 7.87^d^ | 12.04^cd^ | 18.16^bcd^ | 21.75^bc^ | 30.94^ab^ | 1.438 | <0.001 | <0.001 |
| Proteobacteria | 22^abc^ | 17.44^bc^ | 10.88^bc^ | 19.82^abc^ | 37.26^a^ | 27.84^ab^ | 18.27^bc^ | 7.92^c^ | 8.48^c^ | 5.69c | 1.663 | <0.001 | <0.001 |
| Actinobacteria | 0.81^c^ | 1.12^c^ | 1.88^c^ | 7.85^ab^ | 10.94^a^ | 12.62^a^ | 10.59^a^ | 3.09^bc^ | 1.85^c^ | 1.79c | 0.686 | <0.001 | <0.001 |
| Spirochaetae | 2.49^bc^ | 2.52^bc^ | 1.13^c^ | 1.27^c^ | 0.31^c^ | 0.63^c^ | 0.39^c^ | 18.75^a^ | 13.85^ab^ | 10.64^abc^ | 1.105 | <0.001 | <0.001 |
| Tenericutes | 1.03^b^ | 1.16^b^ | 1.49^b^ | 17.95^a^ | 0.64^b^ | 0.56^b^ | 0.54^b^ | 0.55^b^ | 0.7^b^ | 0.84^b^ | 0.774 | <0.001 | <0.001 |
| Unclassified Bacteria | 6.59 | 5.93 | 4.23 | 2.2 | 4.85 | 6.03 | 4.75 | 2.27 | 6.53 | 1.6 | 0.533 | 0.262 | 0.317 |
| Lentisphaerae | 0.16^b^ | 0.29^b^ | 0.65^b^ | 1.06^a^ | 0.25^b^ | 0.48b | 0.47b | 0.28b | 0.22b | 0.31b | 0.047 | <0.001 | <0.001 |
| Acidobacteria | ND | <0.001 | ND | <0.001 | 0.05 | 0.17 | 0.01 | 0.001 | 0.02 | 0.005 | 0.015 | 0.250 | 0.317 |
| Aquificae | ND | ND | ND | ND | ND | 0.001 | ND | ND | ND | ND | 0.000 | 0.452 | 0.506 |
| Armatimonadetes | 0.01 | 0.002 | 0.01 | 0.01 | 0.03 | ND | ND | <0.001 | 0.002 | 0.01 | 0.002 | 0.085 | 0.130 |
| Chlamydiae | ND | ND | ND | ND | ND | <0.001 | 0.03 | 0.002 | ND | ND | 0.002 | 0.225 | 0.305 |
| Chlorobi | ND | <0.001 | ND | ND | 0.01 | 0 | 0.01 | 0.01 | ND | ND | 0.001 | 0.635 | 0.635 |
| Chloroflexi | 0.02 | 0.03 | 0.03 | 0.03 | 0.06 | 0.22 | 0.07 | 0.01 | 0.02 | 0.004 | 0.018 | 0.225 | 0.305 |
| Cyanobacteria | 0.06 | 0.1 | 0.41 | 0.76 | 0.48 | 0.62 | 0.36 | 0.14 | 0.33 | 0.29 | 0.044 | 0.001 | 0.003 |
| Deinococcus-Thermus | 0.01 | 0.07 | 0.005 | 0.01 | 0.28 | 0.21 | 0.03 | 0.01 | 0.01 | 0.003 | 0.023 | 0.045 | 0.074 |
| Elusimicrobia | 0.08 | 0.13 | 0.13 | 0.18 | 0.03 | 0.07 | 0.1 | 0.02 | 0.02 | 0.02 | 0.010 | <0.001 | <0.001 |
| Fibrobacteres | 0.1 | 0.13 | 0.36 | 0.28 | 0.01 | 0.04 | 0.06 | 0.07 | 0.08 | 0.14 | 0.018 | <0.001 | <0.001 |
| Fusobacteria | 0.1 | 0.13 | 0.04 | 0.04 | 0.19 | 0.33 | 0.09 | 0.02 | 0.03 | 0.01 | 0.017 | <0.001 | <0.001 |
| Gemmatimonadetes | ND | ND | ND | ND | ND | 0.03 | ND | ND | 0.004 | 0.004 | 0.003 | 0.462 | 0.506 |
| Planctomycetes | 0.01 | 0.002 | <0.001 | ND | 0.003 | ND | ND | 0.001 | 0.03 | 0.01 | 0.003 | 0.570 | 0.595 |
| Synergistetes | 0.77 | 0.97 | 0.21 | 0.18 | 0.05 | 0.01 | 0.01 | 0.01 | 0.01 | 0.02 | 0.046 | <0.001 | <0.001 |
| Verrucomicrobia | 0.01 | 0.01 | 0.03 | 0.01 | 0.03 | 0.03 | 0.04 | 0.39 | 0.9 | 1.13 | 0.092 | 0.016 | 0.031 |

ND, not detected; ^*^FDR: False discovery rate.

**Table S7**. Comparison of the predominant genera (relative abundance ≥5% in at least one GIT region) in mucosal samples across the gastrointestinal tract of dairy cattle. Mean in the same row with different superscripts represents a significant difference (*P* < 0.05).

| Taxa | Rumen | Reticulum | Omasum | Abomasum | Duodenum | Jejunum | Ileum | Cecum | Colon | Rectum | SEM | P value | FDR^*^ |
| --- | --- | --- | --- | --- | --- | --- | --- | --- | --- | --- | --- | --- | --- |
| Actinobacteria |  |  |  |  |  |  |  |  |  |  |  |  |  |
| Unclassified Bifidobacteriaceae | 0.15^b^ | 0.18^b^ | 0.18^b^ | 6.5^a^ | 1.38^b^ | 1.79^b^ | 1.29^b^ | 0.27^b^ | 0.16^b^ | 0.13^b^ | 0.372 | 0.001 | 0.003 |
| Bacteroidetes |  |  |  |  |  |  |  |  |  |  |  |  |  |
| Unclassified Rikenellaceae | 7.42^ab^ | 8.35^a^ | 6.38^ab^ | 4.79^b^ | 1.54^c^ | 1.2^c^ | 1.15^c^ | 4.99^b^ | 6.3^ab^ | 8.28^a^ | 0.397 | < 0.001 | < 0.001 |
| Unclassified Prevotellaceae | 5.5^a^ | 5.69^a^ | 6.03^a^ | 2.88^bc^ | 1.04^cd^ | 0.46^d^ | 0.48^d^ | 2.01^cd^ | 2.44^bcd^ | 4.18^ab^ | 0.299 | < 0.001 | < 0.001 |
| Unclassified Bacteroidales | 4.82^bc^ | 6.37^ab^ | 8.35^a^ | 5.34^bc^ | 0.91^d^ | 0.95^d^ | 1.11^d^ | 3.49^cd^ | 4.03^bc^ | 6.27^ab^ | 0.359 | < 0.001 | < 0.001 |
| *Prevotella* | 2.95^bc^ | 5.32^bc^ | 17.49^a^ | 6.92^b^ | 1.12^c^ | 1.46^c^ | 0.81^c^ | 0.6^c^ | 1.11^c^ | 1.7^c^ | 0.714 | < 0.001 | < 0.001 |
| Firmicutes |  |  |  |  |  |  |  |  |  |  |  |  |  |
| *Butyrivibrio* | 12.13^a^ | 12.12^a^ | 5.6^b^ | 2.51^bc^ | 3.92^bc^ | 4.15^bc^ | 5.73^b^ | 1.52^c^ | 1.38^c^ | 0.96^c^ | 0.554 | < 0.001 | < 0.001 |
| Unclassified Ruminococcaceae | 7.7^de^ | 8.29^cde^ | 11.82^bcd^ | 8.68^cde^ | 4.92^e^ | 4.04^e^ | 4.3^e^ | 13.52^abc^ | 16.68^ab^ | 18.16^a^ | 0.72 | < 0.001 | < 0.001 |
| Unclassified Lachnospiraceae | 5.77^ab^ | 4.74^abc^ | 4.17^abc^ | 2.82^bc^ | 4.55^abc^ | 6.18^a^ | 5.09^abc^ | 2.26^c^ | 2.87^bc^ | 3.52^abc^ | 0.249 | 0.001 | 0.003 |
| Unclassified Peptostreptococcaceae | 0.22^d^ | 0.04^d^ | 0.05^d^ | 0.04^d^ | 1.63^cd^ | 2.8^bcd^ | 9.78^a^ | 7.36^ab^ | 6.35abc | 5.25abc | 0.538 | < 0.001 | < 0.001 |
| *Turicibacter* | 0.1^b^ | 0.01^b^ | 0.01^b^ | 0.01^b^ | 0.23^b^ | 0.68^b^ | 5.49^a^ | 3.06^ab^ | 2.2^ab^ | 1.78^ab^ | 0.328 | < 0.001 | < 0.001 |
| *Anaerovibrio* | 0.06^b^ | 0.16^b^ | 0.08^b^ | 0.08^b^ | 0.07^b^ | 0.15^b^ | 0.16^b^ | 6.^89a^ | 3.49^ab^ | 2.65^ab^ | 0.407 | < 0.001 | < 0.001 |
| Proteobacteria |  |  |  |  |  |  |  |  |  |  |  |  |  |
| *Campylobacter* | 8.15^a^ | 4.27^b^ | 1.23^c^ | 0.39^c^ | 0.14^c^ | 0.09^c^ | 0.03^c^ | 0.07^c^ | 0.12^c^ | 0.05^c^ | 0.379 | < 0.001 | < 0.001 |
| *Desulfobulbus* | 5.54^a^ | 4.27^b^ | 0.56^c^ | 0.31^c^ | 0.18^c^ | 0.01^c^ | 0^c^ | 0.01^c^ | 0.01^c^ | 0.03^c^ | 0.262 | < 0.001 | < 0.001 |
| *Acinetobacter* | 0.54^d^ | 0.35^d^ | 0.26^d^ | 0.56^d^ | 16.92^a^ | 11.02^ab^ | 8.38^bc^ | 2.54^cd^ | 2.43^cd^ | 1.53^cd^ | 0.837 | < 0.001 | < 0.001 |
| Unclassified Acetobacteraceae | 0.15^b^ | 0.14^b^ | 0.15^b^ | 9.77^a^ | 0.14^b^ | 0.08^b^ | 0.08^b^ | 0.02^b^ | 0.01^b^ | 0.01^b^ | 0.636 | 0.006 | 0.006 |
| *Acetobacter* | 0.1^b^ | 0.09^b^ | 0.09^b^ | 5.28^a^ | 0.06^b^ | 0.06^b^ | 0.13^b^ | 0.02b | ^b^0 | 0.01^b^ | 0.251 | < 0.001 | < 0.001 |
| Spirochaetae |  |  |  |  |  |  |  |  |  |  |  |  |  |
| *Treponema* | 2.23^cd^ | 2.22^cd^ | 1^cd^ | 1.14^cd^ | 0.24^d^ | 0.57^d^ | 0.34^d^ | 16.26^a^ | 12.21^ab^ | 9.13^bcd^ | 1.001 | < 0.001 | < 0.001 |
| Tenericutes |  |  |  |  |  |  |  |  |  |  |  |  |  |
| *Mycoplasma* | 0.2^b^ | 0.19^b^ | 0.19^b^ | 16.66^a^ | 0.17^b^ | 0.01^b^ | 0.03^b^ | 0.01^b^ | 0.01^b^ | 0^b^ | 0.759 | < 0.001 | < 0.001 |

**Table S8**. Comparison of the dominant operational taxonomic units (OTU) (relative abundance ≥2% in at least one region of gastrointestinal tract (GIT)) in mucosal samples across the GIT of dairy cattle. Means in the same row with different superscripts represents a significant difference (*P* < 0.05).

| No. OTU ID2 | Classification | Percentage of total sequences ^1^ | | | | | | | | | | SEM | P value | FDR^*^ |
| --- | --- | --- | --- | --- | --- | --- | --- | --- | --- | --- | --- | --- | --- | --- |
|  |  | Rumen-M | Reticulum-M | Omasum-M | Abomasum-M | Duodenum-M | Jejunum-M | Ileum-M | Cecum-M | Colon-M | Rectum-M |  |  |  |
| OTU5589 | *Acetobacter* | 0.08^b^ | 0.07^b^ | 0.07^b^ | 4.25^a^ | 0.04^b^ | 0.04^b^ | 0.09^b^ | 0.01^b^ | ND | ND | 0.210 | <0.001 | <0.001 |
| OTU332 | *Acinetobacter* | 0.17^c^ | 0.12^c^ | 0.11^c^ | 0.26^c^ | 7.41^a^ | 6.22^a^ | 3.03^b^ | 1.11^bc^ | 1.02^bc^ | 0.67^c^ | 0.361 | <0.001 | <0.001 |
| OTU6869 | *Acinetobacter* | 0.06^b^ | 0.05^b^ | 0.03^b^ | 0.04^b^ | 2.37^a^ | 0.23^b^ | 0.54^b^ | 0.28^b^ | 0.1^b^ | 0.2^b^ | 0.113 | <0.001 | <0.001 |
| OTU4339 | *Anaerovibrio* | 0.01^b^ | <0.001^b^ | <0.001^b^ | <0.001^b^ | 0.04^b^ | 0.14^b^ | 0.14^b^ | 6.18^a^ | 3.21^ab^ | 2.33^ab^ | 0.371 | <0.001 | <0.001 |
| OTU3217 | *Butyrivibrio* | 1.96^a^ | 2.07^a^ | 0.39^b^ | 0.14^b^ | 0.11^b^ | 0.01^b^ | <0.001^b^ | <0.001^b^ | <0.001^b^ | 0.01^b^ | 0.106 | <0.001 | <0.001 |
| OTU1531 | *Butyrivibrio* | 2.56^a^ | 1.65^ab^ | 1.23^b^ | 0.18^c^ | 0.09^c^ | 0.04^c^ | <0.001^c^ | <0.001^c^ | 0.01^c^ | 0.01^c^ | 0.130 | <0.001 | <0.001 |
| OTU2345 | *Campylobacter* | 8.12^a^ | 4.25^b^ | 1.13^c^ | 0.38^c^ | 0.14^c^ | 0.02^c^ | <0.001^c^ | <0.001^c^ | 0.01^c^ | 0.02^c^ | 0.380 | <0.001 | <0.001 |
| OTU87 | *Desulfovibrio* | 0.87^b^ | 2.03^a^ | 0.15^c^ | 0.14^c^ | 0.04^c^ | <0.001^c^ | <0.001^c^ | <0.001^c^ | <0.001^c^ | 0.01^c^ | 0.082 | <0.001 | <0.001 |
| OTU4038 | *Mogibacterium* | 0.33^c^ | 0.49^c^ | 0.42^c^ | 0.51^c^ | 1.65^abc^ | 2.97^a^ | 2.18^ab^ | 0.92^bc^ | 0.52^c^ | 0.72^c^ | 0.140 | <0.001 | <0.001 |
| OTU1306 | *Mycoplasma* | 0.09^b^ | 0.1^b^ | 0.1^b^ | 7.94^a^ | 0.07^b^ | <0.001^b^ | <0.001^b^ | 0.01b | <0.001^b^ | <0.001^b^ | 0.354 | <0.001 | <0.001 |
| OTU1568 | *Mycoplasma* | 0.03^b^ | 0.02^b^ | 0.02^b^ | 2.29^a^ | 0.04^b^ | <0.001^b^ | 0.01^b^ | <0.001b | <0.001^b^ | <0.001^b^ | 0.118 | <0.001 | <0.001 |
| OTU4299 | *Mycoplasma* | 0.03^b^ | 0.03^b^ | 0.03^b^ | 3.19^a^ | 0.03^b^ | <0.001^b^ | <0.001^b^ | ND | <0.001^b^ | <0.001^b^ | 0.177 | <0.001 | <0.001 |
| OTU4097 | *Stenotrophomonas* | 0.03^b^ | 0.03^b^ | 0.03^b^ | 0.06^b^ | 2.1^a^ | 2.25^a^ | 0.57^b^ | 0.41^b^ | 0.42^b^ | 0.22^b^ | 0.133 | <0.001 | <0.001 |
| OTU5108 | *Succiniclasticum* | 1.22^b^ | 1.46^b^ | 2.52^a^ | 1.2^b^ | 0.38^c^ | 0.22^c^ | 0.12^c^ | 0.06^c^ | 0.08^c^ | 0.1^c^ | 0.112 | <0.001 | <0.001 |
| OTU808 | *Treponema* | <0.001^b^ | <0.001^b^ | <0.001^b^ | <0.001^b^ | 0.05^b^ | 0.12^b^ | 0.08^b^ | 3.23^a^ | 3.87^a^ | 2.84^ab^ | 0.273 | <0.001 | <0.001 |
| OTU6044 | *Treponema* | 0.01^b^ | <0.001^b^ | <0.001^b^ | <0.001^b^ | <0.001^b^ | 0.14^b^ | 0.07^b^ | 6.03^a^ | 3.08^ab^ | 1.89^ab^ | 0.402 | 0.002 | 0.001 |
| OTU5368 | *Treponema* | <0.001^b^ | <0.001^b^ | <0.001^b^ | <0.001^b^ | <0.001^b^ | 0.05^b^ | 0.02^b^ | 2.2^a^ | 1.4^ab^ | 0.59^ab^ | 0.161 | 0.007 | 0.007 |
| OTU1317 | *Treponema* | <0.001 | <0.001 | <0.001 | <0.001 | 0.03 | 0.12 | 0.05 | 2.94 | 2.34 | 2.65 | 0.301 | 0.051 | 0.052 |
| OTU1315 | *Turicibacter* | 0.09^b^ | 0.01^b^ | 0.01^b^ | 0.01^b^ | 0.22^b^ | 0.61^b^ | 4.76^a^ | 2.73^ab^ | 2.06^ab^ | 1.61^ab^ | 0.301 | 0.001 | 0.001 |
| OTU2564 | Unclassified Acetobacteraceae | 0.13^b^ | 0.12^b^ | 0.13^b^ | 8.3^a^ | 0.1^b^ | 0.01^b^ | <0.001^b^ | ND | <0.001^b^ | ND | 0.610 | 0.042 | 0.044 |
| OTU1374 | Unclassified Bacteria | 1.46^abc^ | 1.41^abc^ | 1.06^bc^ | 0.47^c^ | 1.05^c^ | 2.36^a^ | 2.21^ab^ | 0.6^c^ | 0.54^c^ | 0.44^c^ | 0.115 | <0.001 | <0.001 |
| OTU5138 | Unclassified Bacteroidales | <0.001^d^ | <0.001^d^ | <0.001^d^ | <0.001^d^ | 0.01^d^ | 0.06^cd^ | 0.22^bcd^ | 0.71^bc^ | 0.77^b^ | 2^a^ | 0.090 | <0.001 | <0.001 |
| OTU5977 | Unclassified Bifidobacteriaceae | 0.13^b^ | 0.15^b^ | 0.14^b^ | 6.07^a^ | 1.11^b^ | 1.46^b^ | 1.06^b^ | 0.24^b^ | 0.12^b^ | 0.1^b^ | 0.338 | <0.001 | <0.001 |
| OTU1956 | Unclassified Lachnospiraceae | 2^a^ | 1.14^b^ | 0.22^c^ | 0.12^c^ | 0.05^c^ | 0.03^c^ | <0.001^c^ | <0.001^c^ | 0.01^c^ | 0.01^c^ | 0.090 | <0.001 | <0.001 |
| OTU6802 | Unclassified Neisseriaceae | 2.07^a^ | 1.76^a^ | 2.01^a^ | 0.34^b^ | 0.06^b^ | 0.01^b^ | <0.001^b^ | <0.001^b^ | 0.01^b^ | 0.02^b^ | 0.141 | <0.001 | <0.001 |
| OTU4916 | Unclassified Peptostreptococcaceae | 0.09^d^ | 0.02^d^ | 0.02^d^ | 0.01^d^ | 0.64^cd^ | 1.33^bcd^ | 3.92^a^ | 2.75^ab^ | 2.73^ab^ | 2.4^abc^ | 0.221 | <0.001 | <0.001 |
| OTU4730 | Unclassified Peptostreptococcaceae | 0.1^d^ | 0.02^d^ | 0.02^d^ | 0.02^d^ | 0.71^cd^ | 1.2^bcd^ | 4.86^a^ | 3.75^ab^ | 3.08^abc^ | 2.33^abcd^ | 0.272 | <0.001 | <0.001 |
| OTU6764 | Unclassified Prevotellaceae | 3.35^a^ | 2.92^ab^ | 2.04^b^ | 0.39^c^ | 0.22^c^ | 0.03^c^ | <0.001^c^ | <0.001^c^ | 0.01^c^ | 0.01^c^ | 0.183 | <0.001 | <0.001 |
| OTU4286 | Unclassified Rhodocyclaceae | 0.64^b^ | 0.82^b^ | 2.86^a^ | 0.3^b^ | 0.06^b^ | <0.001^b^ | <0.001^b^ | <0.001^b^ | <0.001^b^ | 0.02^b^ | 0.135 | <0.001 | <0.001 |
| OTU120 | Unclassified Ruminococcaceae | 1.09^cd^ | 1.4^bc^ | 2.12^a^ | 1.7^ab^ | 0.64^de^ | 0.33^e^ | 0.21^e^ | 0.13^e^ | 0.17^e^ | 0.15^e^ | 0.097 | <0.001 | <0.001 |
| OTU1888 | Unclassified Ruminococcaceae | 0.01^b^ | 0.01^b^ | <0.001^b^ | 0.01^b^ | 0.22^b^ | 0.24^b^ | 0.49^b^ | 2.69^a^ | 3.41^a^ | 3.56^a^ | 0.201 | <0.001 | <0.001 |
| OTU3720 | Unclassified Ruminococcaceae | 0.01^b^ | <0.001^b^ | <0.001^b^ | <0.001^b^ | 0.09^b^ | 0.11^b^ | 0.24^b^ | 1.91^a^ | 2.01^a^ | 1.9^a^ | 0.121 | <0.001 | <0.001 |
| OTU5821 | Unclassified Spirochaetaceae | <0.001^b^ | <0.001^b^ | <0.001^b^ | <0.001^b^ | 0.02^b^ | 0.06^b^ | 0.05^b^ | 2.46^a^ | 1.58^a^ | 1.47^a^ | 0.202 | 0.018 | 0.0193 |

^1^Values represent means.

^2^A total of 6860 OTUs were numbered in serial order.

**Table S9.** Analysis of molecular variation hypothesis testing results in determining the difference in structure of the bacterial population between the digesta and its corresponding mucosal samples. The star symbol indicates there is a significant difference in the structure of the bacterial population between the digesta and its corresponding mucosal tissues.

| Samples types | | *P* value |
| --- | --- | --- |
| Digesta | Mucosa |
| Rumen | Rumen | < 0.001* |
| Reticulum | Reticulum | < 0.001* |
| Omasum | Omasum | < 0.001* |
| Abomasum | Abomasum | < 0.001* |
| Duodenum | Duodenum | < 0.001* |
| Jejunum | Jejunum | < 0.001* |
| Ileum | Ileum | < 0.001* |
| Cecum | Cecum | < 0.001* |
| Colon | Colon | < 0.001* |
| Rectum | Rectum | < 0.001* |

**Table S10**. Predicted functions of the digesta-assoicated bacterial microbiota throughout the GIT of dairy cattle. Means with same superscript within the same row are not significantly different at P < 0.05.

| Functions | Rumen | Reticulum | Abomasum | Omasum | Duodenum | Jejunum | Ileum | Cecum | Colon | Rectum | SEM | P value | FDR^*^ |
| --- | --- | --- | --- | --- | --- | --- | --- | --- | --- | --- | --- | --- | --- |
| **Cellular Processes** |  |  |  |  |  |  |  |  |  |  |  |  |  |
| Cell Motility | 3.2^bcd^ | 3.08^bcd^ | 3.07^bcd^ | 2.99^d^ | 3.16^bcd^ | 3.01^cd^ | 3.16^bcd^ | 3.29^bc^ | 3.37^b^ | 3.73^a^ | 0.023 | <0.001 | <0.001 |
| Transport and Catabolism | 0.46^a^ | 0.4^b^ | 0.42^ab^ | 0.25^c^ | 0.17^d^ | 0.17^d^ | 0.16^d^ | 0.16^d^ | 0.16^d^ | 0.16^d^ | 0.063 | <0.001 | <0.001 |
| Cell Growth and Death | 0.31^bcd^ | 0.31^bc^ | 0.31^bc^ | 0.4^a^ | 0.28^cde^ | 0.34^b^ | 0.28^de^ | 0.27^e^ | 0.28^de^ | 0.25^e^ | 0.025 | <0.001 | <0.001 |
| **Environmental Information Processing** |  |  |  |  |  |  |  |  |  |  |  |  |  |
| Membrane Transport | 17.08^cd^ | 16.84^cd^ | 16.14^d^ | 16.12^d^ | 16.99^cd^ | 16.86^cd^ | 17.36^cd^ | 18.57^bc^ | 19.37^b^ | 22.89^a^ | 0.458 | <0.001 | <0.001 |
| Signal Transduction | 1.82^d^ | 1.92^d^ | 1.91^d^ | 1.92^d^ | 2.8^a^ | 2.39^bc^ | 2.72^ab^ | 2.61^ab^ | 2.44^b^ | 2.1^cd^ | 0.001 | <0.001 | <0.001 |
| Signaling Molecules and Interaction | 0.07^c^ | 0.08^c^ | 0.08^c^ | 0.1^b^ | 0.12^a^ | 0.12^a^ | 0.12^a^ | 0.12^a^ | 0.11^ab^ | 0.08^b^ | 0.007 | <0.001 | <0.001 |
| **Genetic Information Processing** |  |  |  |  |  |  |  |  |  |  |  |  |  |
| Replication and Repair | 8.8^a^ | 8.61^a^ | 8.65^a^ | 8.64^a^ | 6.11^d^ | 7.31^bc^ | 6.32^cd^ | 6.72^bcd^ | 7.34^b^ | 8.56^a^ | 0.047 | <0.001 | <0.001 |
| Translation | 3.81^b^ | 3.94^b^ | 4^b^ | 4.66^a^ | 3.47^c^ | 4.07^b^ | 3.46^c^ | 3.34^cd^ | 3.4^c^ | 3^d^ | 0.089 | <0.001 | <0.001 |
| Transcription | 3.78^b^ | 3.68^bc^ | 3.46^bcd^ | 3.36^bcd^ | 3.07^d^ | 3.17^cd^ | 3.22^cd^ | 3.55^bcd^ | 3.87^b^ | 4.9^a^ | 0.006 | <0.001 | <0.001 |
| Folding, Sorting and Degradation | 1.97^c^ | 2.03^abc^ | 2.06^abc^ | 2.08^abc^ | 2.11^a^ | 2.1^ab^ | 2.09^ab^ | 2.03^abc^ | 1.99^bc^ | 1.81^d^ | 0.001 | <0.001 | <0.001 |
| **Human Diseases** |  |  |  |  |  |  |  |  |  |  |  |  |  |
| Infectious Diseases | 0.3^d^ | 0.33^cd^ | 0.34^cd^ | 0.33^cd^ | 0.56^a^ | 0.45^ab^ | 0.53^ab^ | 0.48^ab^ | 0.43^bc^ | 0.28^d^ | 0.033 | <0.001 | <0.001 |
| Metabolic Diseases | 0.06^cd^ | 0.07^bc^ | 0.07^bc^ | 0.08^a^ | 0.06^c^ | 0.08^ab^ | 0.06^c^ | 0.06^cd^ | 0.06^c^ | 0.05^d^ | 0.006 | <0.001 | <0.001 |
| Cancers | 0.05^d^ | 0.06^d^ | 0.06^d^ | 0.08^c^ | 0.1^a^ | 0.1^abc^ | 0.1^ab^ | 0.09^abc^ | 0.08^bc^ | 0.06^d^ | 0.008 | <0.001 | <0.001 |
| Neurodegenerative Diseases | 0.04^c^ | 0.05^c^ | 0.05^c^ | 0.06^c^ | 0.18^a^ | 0.13^b^ | 0.17^ab^ | 0.15^ab^ | 0.13^b^ | 0.08^c^ | 0.050 | <0.001 | <0.001 |
| Immune System Diseases | 0.01^d^ | 0.02^d^ | 0.02^d^ | 0.02^cd^ | 0.04^a^ | 0.03^bc^ | 0.04^ab^ | 0.03^ab^ | 0.03^bc^ | 0.02^d^ | 0.002 | <0.001 | <0.001 |
| **Metabolism** |  |  |  |  |  |  |  |  |  |  |  |  |  |
| Amino Acid Metabolism | 8.38^bcd^ | 8.46^bc^ | 8.62^b^ | 9.02^a^ | 8.3^cd^ | 8.59^b^ | 8.26^cd^ | 8.14^d^ | 8.1^d^ | 7.72^e^ | 0.023 | <0.001 | <0.001 |
| Energy Metabolism | 4.9^a^ | 4.89^a^ | 4.92^a^ | 5^a^ | 4.6^bc^ | 4.7^b^ | 4.58^bc^ | 4.52^c^ | 4.47^cd^ | 4.32^d^ | 0.033 | <0.001 | <0.001 |
| Metabolism of Cofactors and Vitamins | 3.23^de^ | 3.3^cd^ | 3.38^abcd^ | 3.47^abc^ | 3.55^a^ | 3.44^abcd^ | 3.53^ab^ | 3.43^abcd^ | 3.31^bcd^ | 3.01^e^ | 0.002 | <0.001 | <0.001 |
| Lipid Metabolism | 3.1^a^ | 3.08^a^ | 3.04^abc^ | 3.07^ab^ | 2.99^bcd^ | 2.99^cde^ | 2.98^cde^ | 2.95^de^ | 2.95^de^ | 2.92^e^ | 0.030 | <0.001 | <0.001 |
| Nucleotide Metabolism | 2.99^bc^ | 3.04^bc^ | 3.11^b^ | 3.34^a^ | 2.9^cd^ | 3.17^ab^ | 2.87^cd^ | 2.77^d^ | 2.73^d^ | 2.35^e^ | 0.003 | <0.001 | <0.001 |
| Glycan Biosynthesis and Metabolism | 2.58^ab^ | 2.49^abc^ | 2.61^a^ | 1.96^ef^ | 2.42^abcd^ | 2.13^cde^ | 2.36^abcd^ | 2.23^bcde^ | 2.07^def^ | 1.74^f^ | 0.024 | <0.001 | <0.001 |
| Enzyme Families | 2.48^a^ | 2.44^ab^ | 2.48^a^ | 2.34^b^ | 2.13^cd^ | 2.22^c^ | 2.11^d^ | 2.1^d^ | 2.06^de^ | 1.97^e^ | 0.000 | <0.001 | <0.001 |
| Metabolism of Terpenoids and Polyketides | 2.05^a^ | 1.94^a^ | 1.93^a^ | 1.8^b^ | 1.48^c^ | 1.58^c^ | 1.51^c^ | 1.53^c^ | 1.58^c^ | 1.72^b^ | 0.010 | <0.001 | <0.001 |
| Carbohydrate Metabolism | 11.22^ab^ | 11.23^ab^ | 11.39^a^ | 10.94^bc^ | 10.7^cd^ | 10.86^bc^ | 10.6^cd^ | 10.35^de^ | 10.13^e^ | 9.33^f^ | 0.006 | <0.001 | <0.001 |
| Xenobiotics Biodegradation and Metabolism | 1.53^d^ | 1.59^d^ | 1.56^d^ | 1.82^c^ | 2.12^a^ | 2.08^ab^ | 2.14^a^ | 2.09^ab^ | 2.04^ab^ | 1.92^bc^ | 0.014 | <0.001 | <0.001 |
| Metabolism of Other Amino Acids | 1.27^de^ | 1.29^cde^ | 1.34^cde^ | 1.3^cde^ | 1.79^a^ | 1.55^abc^ | 1.74^a^ | 1.61^ab^ | 1.45^bcd^ | 1.08^e^ | 0.009 | <0.001 | <0.001 |
| Biosynthesis of Other Secondary Metabolites | 1.04^a^ | 0.99^a^ | 1.04a | 0.88^b^ | 0.68^cd^ | 0.73^c^ | 0.66^cd^ | 0.64^cd^ | 0.64^cd^ | 0.6^d^ | 0.280 | <0.001 | <0.001 |
| **Organismal Systems** |  |  |  |  |  |  |  |  |  |  |  |  |  |
| Endocrine System | 0.62^a^ | 0.55^ab^ | 0.53^b^ | 0.41^c^ | 0.16^e^ | 0.24^de^ | 0.16^e^ | 0.18^de^ | 0.22^de^ | 0.26^d^ | 0.001 | <0.001 | <0.001 |
| Nervous System | 0.1^ab^ | 0.1^ab^ | 0.09^abc^ | 0.11^a^ | 0.05^e^ | 0.07^bcd^ | 0.05^e^ | 0.05^de^ | 0.06^de^ | 0.07^cde^ | 0.003 | <0.001 | <0.001 |
| Environmental Adaptation | 0.14^b^ | 0.14^b^ | 0.14^b^ | 0.18^a^ | 0.11^c^ | 0.15^b^ | 0.11^c^ | 0.11^c^ | 0.11^c^ | 0.1^c^ | 0.027 | <0.001 | <0.001 |
| Immune System | 0.05^ef^ | 0.05^de^ | 0.05^de^ | 0.07^a^ | 0.06^bc^ | 0.07^ab^ | 0.06^bc^ | 0.06^cd^ | 0.06^cd^ | 0.05^f^ | 0.146 | <0.001 | <0.001 |
| Digestive System | 0.03^d^ | 0.04^cd^ | 0.04^cd^ | 0.04^bc^ | 0.06^a^ | 0.06^a^ | 0.06^a^ | 0.05^ab^ | 0.05^ab^ | 0.03^cd^ | 0.003 | <0.001 | <0.001 |
| Excretory System | 0.01^c^ | 0.02^bc^ | 0.02^bc^ | 0.02^ab^ | 0.02^a^ | 0.02^a^ | 0.02^a^ | 0.02^ab^ | 0.02^ab^ | 0.01^c^ | 0.073 | <0.001 | <0.001 |
| **Unclassified** |  |  |  |  |  |  |  |  |  |  |  |  |  |
| Poorly Characterized | 4.45^ef^ | 4.55^ef^ | 4.6de | 4.58^de^ | 5.53^a^ | 5.1^abc^ | 5.43^a^ | 5.15^ab^ | 4.86^bcd^ | 4.01^f^ | 0.002 | <0.001 | <0.001 |
| Cellular Processes and Signaling | 3.83^d^ | 3.98^d^ | 4.05^cd^ | 3.9^d^ | 5.44^a^ | 4.69^bc^ | 5.35^ab^ | 5.06^ab^ | 4.72^bc^ | 3.97^d^ | 0.081 | <0.001 | <0.001 |
| Metabolism | 2.19^f^ | 2.33^f^ | 2.29^f^ | 2.43^ef^ | 3.43^a^ | 2.99^cd^ | 3.38^ab^ | 3.27^abc^ | 3.07^bc^ | 2.69^de^ | 0.013 | <0.001 | <0.001 |
| Genetic Information Processing | 2.03^c^ | 2.11^b^ | 2.13^b^ | 2.23^a^ | 2.24^a^ | 2.24^a^ | 2.25^a^ | 2.22^a^ | 2.22^a^ | 2.15^b^ | 0.042 | <0.001 | <0.001 |

^*^FDR: False discovery rate.

**Table S11.** Comparisons of the predicted functions of the mucosa-assoicated bacterial microbiota throughout the GIT of dairy cattle. Means with same superscript within the same row are not significantly different at *P* < 0.05.

| Mucosa | Rumen | Reticulum | Abomasum | Omasum | Duodenum | Jejunum | Ileum | Cecum | Colon | Rectum | SEM | P value | FDR^*^ |  |
| --- | --- | --- | --- | --- | --- | --- | --- | --- | --- | --- | --- | --- | --- | --- |
| **Cellular Processes** |  |  |  |  |  |  |  |  |  |  |  |  |  |  |
| Cell Growth and Death | 0.32^bcde^ | 0.31^de^ | 0.32^cde^ | 0.38^abc^ | 0.38^abcd^ | 0.4^a^ | 0.39^ab^ | 0.34^abcde^ | 0.32^bcde^ | 0.3^e^ | 0.006 | <0.001 | <0.001 |  |
| Cell Motility | 4.17^ab^ | 3.95^abc^ | 3.09^c^ | 2.95^c^ | 3.18^bc^ | 3.05^c^ | 3.02^c^ | 4.8^a^ | 4.43^a^ | 4.32^a^ | 0.109 | <0.001 | <0.001 |  |
| Transport and Catabolism | 0.36^bc^ | 0.4^abc^ | 0.45^ab^ | 0.34^bc^ | 0.27^c^ | 0.25^c^ | 0.25^c^ | 0.45^ab^ | 0.49^ab^ | 0.54^a^ | 0.016 | <0.001 | <0.001 |  |
| **Environmental Information Processing** |  |  |  |  |  |  |  |  |  |  |  |  |  |  |
| Membrane Transport | 17.82^abc^ | 17.73^abc^ | 16.33^c^ | 16.72^c^ | 16.3^c^ | 16.8^c^ | 17.1^bc^ | 19.8^a^ | 19.18^ab^ | 18.28^abc^ | 0.201 | <0.001 | <0.001 |  |
| Signal Transduction | 2.62^a^ | 2.44^ab^ | 1.86^d^ | 1.8^d^ | 2.28^abc^ | 2.13^bcd^ | 2.02^cd^ | 2.47^ab^ | 2.33^abc^ | 2.16^bcd^ | 0.041 | <0.001 | <0.001 |  |
| Signaling Molecules and Interaction | 0.08^bc^ | 0.08^bc^ | 0.08^bc^ | 0.11^ab^ | 0.14^a^ | 0.14^a^ | 0.13^a^ | 0.07^c^ | 0.07^bc^ | 0.06^c^ | 0.004 | <0.001 | <0.001 |  |
| **Genetic Information Processin**g |  |  |  |  |  |  |  |  |  |  |  |  |  |  |
| Folding, Sorting and Degradation | 1.83^a^ | 1.85^a^ | 1.96^a^ | 1.74^a^ | 1.66^a^ | 1.82^a^ | 1.88^a^ | 1.69^a^ | 1.7^a^ | 1.85^a^ | 0.022 | 0.082 | 0.091 |  |
| Replication and Repair | 7.59^abc^ | 7.79^abc^ | 8.41^ab^ | 8.73^a^ | 6.66^c^ | 7.11^c^ | 7.73^abc^ | 7.21^bc^ | 7.22^bc^ | 7.74^abc^ | 0.111 | <0.001 | <0.001 |  |
| Transcription | 3.01^d^ | 3.06^d^ | 3.53^abc^ | 3.26^bcd^ | 2.91^d^ | 2.98^d^ | 3.22^cd^ | 3.52^abc^ | 3.61^ab^ | 3.65^a^ | 0.042 | <0.001 | <0.001 |  |
| Translation | 3.31^ab^ | 3.37^ab^ | 3.91^ab^ | 3.89^ab^ | 3.32^ab^ | 3.74^ab^ | 3.95^a^ | 3.28^ab^ | 3.11^b^ | 3.24a^b^ | 0.064 | <0.001 | <0.001 |  |
| **Human Diseases** |  |  |  |  |  |  |  |  |  |  |  |  |  |  |
| Cancers | 0.04^b^ | 0.04^b^ | 0.05^b^ | 0.06^b^ | 0.12^a^ | 0.12^a^ | 0.1^a^ | 0.06^b^ | 0.06^b^ | 0.05^b^ | 0.004 | 0.294 | 0.310 |  |
| Cardiovascular Diseases | 0^c^ | 0c | 0c | 0c | 0.02^a^ | 0.02^ab^ | 0.01^abc^ | 0.01^bc^ | 0.01^bc^ | 0^c^ | 0.001 | 0.002 | 0.003 |  |
| Immune System Diseases | 0.01^a^ | 0.01^a^ | 0.01^a^ | 0.02a | 0.02^a^ | 0.02^a^ | 0.02a | 0.01^a^ | 0.01a | 0.02^a^ | 0.001 | <0.001 | <0.001 |  |
| Infectious Diseases | 0.29^bcd^ | 0.28^bcd^ | 0.28^d^ | 0.28^cd^ | 0.35a | 0.36^a^ | 0.32abcd | 0.36^a^ | 0.34^abc^ | 0.34^ab^ | 0.005 | <0.001 | <0.001 |  |
| Metabolic Diseases | 0.04^d^ | 0.04^cd^ | 0.06^abc^ | 0.06^ab^ | 0.07a | 0.07^a^ | 0.07^a^ | 0.04^bcd^ | 0.04^bcd^ | 0.04^bcd^ | 0.002 | <0.001 | <0.001 |  |
| Neurodegenerative Diseases | 0.08^bcd^ | 0.07^cd^ | 0.04^d^ | 0.06^cd^ | 0.17a | 0.15^ab^ | 0.13^abc^ | 0.08^bcd^ | 0.09^bcd^ | 0.07^cd^ | 0.007 | <0.001 | <0.001 |  |
| **Metabolism** |  |  |  |  |  |  |  |  |  |  |  |  | <0.001 |  |
| Amino Acid Metabolism | 8.89^bc^ | 8.86^bc^ | 8.87^bc^ | 8.52^c^ | 10.2^a^ | 9.84^ab^ | 9.54^abc^ | 8.71^c^ | 9^bc^ | 8.89^bc^ | 0.095 | <0.001 | <0.001 |  |
| Biosynthesis of Other Secondary Metabolites | 0.85^cd^ | 0.93^bc^ | 1.05^a^ | 1.01^ab^ | 0.86^cd^ | 0.85^cd^ | 0.82^d^ | 0.84^cd^ | 0.92^bcd^ | 1.05^a^ | 0.013 | <0.001 | <0.001 |  |
| Carbohydrate Metabolism | 10.76^abc^ | 10.8^abc^ | 11.31^a^ | 11.21^a^ | 10.95^ab^ | 10.87^ab^ | 10.63^bc^ | 10.26^c^ | 10.53^bc^ | 10.6^bc^ | 0.052 | <0.001 | <0.001 |  |
| Energy Metabolism | 5.1^ab^ | 5.15^a^ | 4.96^abc^ | 4.83^abc^ | 4.65^c^ | 4.84^abc^ | 4.8^abc^ | 4.68^c^ | 4.67^c^ | 4.77^bc^ | 0.032 | <0.001 | <0.001 |  |
| Enzyme Families | 2.4^ab^ | 2.44^ab^ | 2.41^ab^ | 2.51^a^ | 1.98^d^ | 2.07^cd^ | 2.07^cd^ | 2.09^cd^ | 2.12^cd^ | 2.28^bc^ | 0.028 | <0.001 | <0.001 |  |
| Glycan Biosynthesis and Metabolism | 2.08^abc^ | 2.22^ab^ | 2.45^a^ | 2.13^ab^ | 1.6^c^ | 1.62^c^ | 1.6^c^ | 1.92^bc^ | 2.13^ab^ | 2.51^a^ | 0.051 | <0.001 | <0.001 |  |
| Lipid Metabolism | 3.5^ab^ | 3.45^b^ | 3.46^b^ | 3.52^ab^ | 4.24^a^ | 3.79^ab^ | 3.6^ab^ | 3.16^b^ | 3.43^b^ | 3.28b | 0.060 | <0.001 | <0.001 |  |
| Metabolism of Cofactors and Vitamins | 3.6^ab^ | 3.56^ab^ | 3.4^bc^ | 3.73^a^ | 3.42^bc^ | 3.46^ab^ | 3.37^bcd^ | 3.08^d^ | 3.14^cd^ | 3.31bcd | 0.031 | 0.337 | 0.344 |  |
| Metabolism of Other Amino Acids | 1.37^bc^ | 1.37^bc^ | 1.4^bc^ | 1.47^bc^ | 2^a^ | 1.79^ab^ | 1.65^abc^ | 1.35^bc^ | 1.38^bc^ | 1.29^c^ | 0.039 | 0.003 | 0.004 |  |
| Metabolism of Terpenoids and Polyketides | 2.06^b^ | 2.13^ab^ | 2.18^ab^ | 2.17^ab^ | 2.5^a^ | 2.22^ab^ | 2.2a^b^ | 2.1^ab^ | 2.2^ab^ | 2.13^ab^ | 0.031 | <0.001 | <0.001 |  |
| Nucleotide Metabolism | 2.66^bcde^ | 2.68^bcde^ | 2.98^abcd^ | 3.15^a^ | 2.7^bcde^ | 2.99^abc^ | 3.05^ab^ | 2.57^de^ | 2.46^e^ | 2.59^cde^ | 0.039 | 0.007 | 0.008 |  |
| Xenobiotics Biodegradation and Metabolism | 2.28^bc^ | 2.16^bc^ | 1.8^bc^ | 2.12^bc^ | 3.63^a^ | 3.1^ab^ | 2.96^ab^ | 1.89^bc^ | 2.02^bc^ | 1.6^c^ | 0.116 | 0.004 | 0.005 |  |
| **Organismal Systems** |  |  |  |  |  |  |  |  |  |  |  |  |  |  |
| Circulatory System | 0.001 | 0.001 | 0.001 | 0.002 | 0.002 | 0.001 | 0.001 | 0.001 | 0.002 | 0.002 | 0.000 | 0.517 | 0.552 |  |
| Digestive System | 0.01^d^ | 0.01d | 0.03abc | 0.03^abc^ | 0.03^ab^ | 0.03^a^ | 0.03^a^ | 0.02b^cd^ | 0.02^cd^ | 0.01^d^ | 0.001 | <0.001 | <0.001 |  |
| Endocrine System | 0.46^bc^ | 0.51^ab^ | 0.63^a^ | 0.61^a^ | 0.38^c^ | 0.36^c^ | 0.36^c^ | 0.57^ab^ | 0.58ab | 0.6^a^ | 0.016 | <0.001 | <0.001 |  |
| Environmental Adaptation | 0.19^ab^ | 0.2^ab^ | 0.15^ab^ | 0.14^b^ | 0.16^ab^ | 0.16^ab^ | 0.17^ab^ | 0.21^a^ | 0.17^ab^ | 0.16^ab^ | 0.004 | 0.116 | 0.125 |  |
| Excretory System | 0^b^ | 0.01^b^ | 0.01^a^ | 0.01^a^ | 0.01^a^ | 0.01^a^ | 0.01^a^ | 0.01^a^ | 0.01^ab^ | 0.01^b^ | 0.000 | <0.001 | <0.001 |  |
| Immune System | 0.03b | 0.03b | 0.04^ab^ | 0.05^ab^ | 0.05^ab^ | 0.05^a^ | 0.06^a^ | 0.03^b^ | 0.03^b^ | 0.03^b^ | 0.002 | <0.001 | <0.001 |  |
| Nervous System | 0.1b | 0.1b | 0.11^ab^ | 0.16^a^ | 0.11^ab^ | 0.12^ab^ | 0.11^b^ | 0.11^ab^ | 0.12^ab^ | 0.12^ab^ | 0.004 | 0.010 | 0.011 |  |
| Unclassified |  |  |  |  |  |  |  |  |  |  |  |  |  |  |
| Cellular Processes and Signaling | 3.72^a^ | 3.68^a^ | 3.87^a^ | 3.69^a^ | 4.02^a^ | 3.85^a^ | 3.81^a^ | 3.69^a^ | 3.75^a^ | 3.85^a^ | 0.032 | 0.344 | 0.352 |  |
| Genetic Information Processing | 1.82^b^ | 1.83^b^ | 1.91^ab^ | 1.99^ab^ | 1.86^ab^ | 1.96^ab^ | 2.01^ab^ | 2.06^a^ | 1.98^ab^ | 2a^b^ | 0.017 | <0.001 | <0.001 |  |
| Metabolism | 2.3^abc^ | 2.2^bc^ | 2.2^bc^ | 2.24^abc^ | 2.5^a^ | 2.47^ab^ | 2.48^a^ | 2.11^c^ | 2.14^c^ | 2.07^c^ | 0.026 | 0.032 | 0.036 |  |
| Poorly Characterized | 4.24^a^ | 4.26^a^ | 4.38^a^ | 4.3^a^ | 4.33^a^ | 4.39^a^ | 4.36^a^ | 4.32^a^ | 4.19^a^ | 4.2^a^ | 0.020 | <0.001 | <0.001 |  |

^*^FDR: False discovery rate.

**Table S12**. Comparisons of the gene abundance assigned to major pathways (only the gene which relative abundance ≥5% in at least one GIT region were presented) inferred from 16S rRNA gene sequence information in the digesta and mucosal samples using PICRUSt. Means with different superscript within the same row are significantly different at P < 0.05.

|  | Rumen | Reticulum | Abomasum | Omasum | Duodenum | Jejunum | Ileum | Cecum | Colon | Rectum | SEM | P value | FDR^*^ |
| --- | --- | --- | --- | --- | --- | --- | --- | --- | --- | --- | --- | --- | --- |
| Digesta |  |  |  |  |  |  |  |  |  |  |  |  |  |
| Membrane Transport | 17.08^cd^ | 16.84^cd^ | 16.14^d^ | 16.12^d^ | 16.99^cd^ | 16.86^cd^ | 17.36^cd^ | 18.57^bc^ | 19.37^b^ | 22.89^a^ | 0.458 | <0.001 | <0.001 |
| Carbohydrate Metabolism | 11.22^ab^ | 11.23^ab^ | 11.39 ^a^ | 10.94^bc^ | 10.7^cd^ | 10.86^bc^ | 10.6^cd^ | 10.35^de^ | 10.13^e^ | 9.33^f^ | 0.006 | <0.001 | <0.001 |
| Replication and Repair | 8.8^a^ | 8.61^a^ | 8.65^a^ | 8.64^a^ | 6.11^d^ | 7.31^bc^ | 6.32^cd^ | 6.72^bcd^ | 7.34^b^ | 8.56^a^ | 0.047 | <0.001 | <0.001 |
| Amino Acid Metabolism | 8.38^bcd^ | 8.46^bc^ | 8.62 ^b^ | 9.02 ^a^ | 8.3^cd^ | 8.59^b^ | 8.26^cd^ | 8.14^d^ | 8.1^d^ | 7.72^e^ | 0.023 | <0.001 | <0.001 |
| Energy Metabolism | 4.9^a^ | 4.89^a^ | 4.92^a^ | 5^a^ | 4.6^bc^ | 4.7^b^ | 4.58^bc^ | 4.52^c^ | 4.47^cd^ | 4.32^d^ | 0.033 | <0.001 | <0.001 |
| Mucosa |  |  |  |  |  |  |  |  |  |  |  |  |  |
| Membrane Transport | 17.82^abc^ | 17.73^abc^ | 16.33^c^ | 16.72^c^ | 16.3^c^ | 16.8^c^ | 17.1^bc^ | 19.8^a^ | 19.18ab | 18.28^abc^ | 0.052 | <0.001 | <0.001 |
| Carbohydrate Metabolism | 10.76^abc^ | 10.8^abc^ | 11.31^a^ | 11.21^a^ | 10.95^ab^ | 10.87^ab^ | 10.63^bc^ | 10.26^c^ | 10.53^bc^ | 10.6^bc^ | 0.001 | <0.001 | <0.001 |
| Amino Acid Metabolism | 8.89^bc^ | 8.86^bc^ | 8.87^bc^ | 8.52^c^ | 10.2^a^ | 9.84^ab^ | 9.54^abc^ | 8.71^c^ | 9bc | 8.89^bc^ | 9.513 | <0.001 | <0.001 |
| Replication and Repair | 7.59^abc^ | 7.79^abc^ | 8.41^ab^ | 8.73^a^ | 6.66^c^ | 7.11^c^ | 7.73^abc^ | 7.21bc | 7.22^bc^ | 7.74^abc^ | 0.013 | <0.001 | <0.001 |
| Energy Metabolism | 5.1^ab^ | 5.15^a^ | 4.96^abc^ | 4.83^abc^ | 4.65^c^ | 4.84^abc^ | 4.8^abc^ | 4.68^c^ | 4.67^c^ | 4.77^bc^ | 0.005 | <0.001 | <0.001 |

^*^FDR: False

**Table S13**. Ingredients and nutrients of the experimental diets

| Ingredients | Composition (%) | Nutrients | Contents |
| --- | --- | --- | --- |
| Ground corn grain | 27.0 | Dry matter (DM), % | 52.9 |
| Wheat bran | 5.1 | Organic matter, % of DM | 92.0 |
| Soybean meal | 12.7 | Crude protein, % of DM | 16.7 |
| Cottonseed meal | 4.3 | Neutral detergent fiber, % of DM | 31.1 |
| Beet pulp | 1.0 | Acid detergent fiber, % of DM | 18.9 |
| Corn silage | 15.0 | Non-fiber carbohydrate, % of DM | 40.6 |
| Alfalfa hay | 23.0 | Calcium, % of DM | 0.91 |
| Chinese wild grass hay | 7.0 | Phosphorus, % of DM | 0.47 |
| Corn stover | 0.0 | Lysine, % of DM | 0.74 |
| Rice straw | 0.0 | Methionine, % of DM | 0.12 |
| Urea | 0.0 | Net energy actation , Mcal/kg | 1.57 |
| Premix^1^ | 4.9 |  |  |

1: Formulated to provide (per kg of dry matter) 174 g of zeolite powder, 1.25 g of yeast, 25 g of mold removal agent, 21.44 g of KCl, 41.25 g of MgO, 150 g of Salt, 187.5 g of NaHCO3, 84 g of Ca, 15 g of P, 125,000 IU of vitamin A, 750,000 IU of vitamin D3, 937.5 IU of vitamin E, 1750 mg of Zn, 17.5 mg of Se, 28.75 mg of I, 375 mg of Fe, 15 mg of Co, 556.5 mg of Mn and 343.75 mg of Cu. Water content ≤ 10%.
